# YTHDF2-Mediated m6A methylation inhibition by miR27a as a protective mechanism against hormonal osteonecrosis in BMSCs

**DOI:** 10.1186/s12891-024-07481-3

**Published:** 2024-05-06

**Authors:** Tianyi Yuan, Hongjiang Liu, Maimaitiyibubaji Abudoukadier, Zengqiang Yang, Zhiheng Zhou, Yong Cui

**Affiliations:** 1 The Fifth Clinical Medical College of Xinjiang Medical University, Xinjiang Uygur Autonomous Region, Urumqi, 830011 China; 2 Department of Orthopedic Center, The Fifth Affiliated Hospital of Xinjiang Medical University, Xinjiang Uygur Autonomous Region, Urumqi, 830011 China

**Keywords:** Steroid-induced necrosis of the femoral head, Bone marrow mesenchymal stem cells, miRNA, N6-methyladenosine

## Abstract

**Background:**

With the increasing incidence of steroid-induced necrosis of the femoral head (SNFH), numerous scholars have investigated its pathogenesis. Current evidence suggests that the imbalance between lipogenesis and osteoblast differentiation in bone marrow mesenchymal stem cells (BMSCs) is a key pathological feature of SNFH. MicroRNAs (miRNAs) have strong gene regulatory effects and can influence the direction of cell differentiation. N6-methyladenosine (m6A) is a prevalent epigenetic modification involved in diverse pathophysiological processes. However, knowledge of how miRNAs regulate m6A-related factors that affect BMSC differentiation is limited.

**Objective:**

We aimed to investigate the role of miR27a in regulating the expression of YTHDF2 in BMSCs.

**Methods:**

We compared miR27a, YTHDF2, and total m6A mRNA levels in SNFH-affected and control BMSCs. CCK-8 and TUNEL assays were used to assess BMSC proliferation and apoptosis. Western blotting and qRT‒PCR were used to measure the expression of osteogenic (ALP, RUNX2, and OCN) and lipogenic (PPARγ and C/EBPα) markers. Alizarin Red and Oil Red O staining were used to quantify osteogenic and lipogenic differentiation, respectively. miR27a was knocked down or overexpressed to evaluate its impact on BMSC differentiation and its relationship with YTHDF2. Bioinformatics analyses identified YTHDF2 as a differentially expressed gene in SNFH (ROC analysis) and revealed potential signaling pathways through GSEA. The effects of YTHDF2 silencing on the lipogenic and osteogenic functions of BMSCs were assessed.

**Results:**

miR27a downregulation and YTHDF2 upregulation were observed in the SNFH BMSCs. miR27a knockdown/overexpression modulated YTHDF2 expression, impacting BMSC differentiation. miR27a silencing decreased m6A methylation and promoted osteogenic differentiation, while YTHDF2 silencing exerted similar effects. GSEA suggested potential signaling pathways associated with YTHDF2 in SNFH.

**Conclusion:**

miR27a regulates BMSC differentiation through YTHDF2, affecting m6A methylation and promoting osteogenesis. This finding suggests a potential therapeutic target for SNFH.

**Supplementary Information:**

The online version contains supplementary material available at 10.1186/s12891-024-07481-3.

## Introduction

Necrosis of the femoral head is a pathological condition with multiple causes that substantially impacts quality of life [1]. In the USA and Europe, 2–10% of hip replacements are attributed to this condition, while in Asia, this figure is 60% [2]. Among nontraumatic cases, steroid-induced necrosis of the femoral head (SNFH) has the highest incidence and is steadily increasing [3]. According to the latest statistics, without effective intervention, 80% of patients with hormonal femoral head necrosis experience collapse of the femoral head, leading to loss of femoral head function and seriously impacting the patient’s quality of life [4]. SNFH often occurs after heavy, long-term use of hormonal drugs, but its exact pathogenesis remains unclear [5]. To date, numerous studies have been conducted on SNFH from both epigenetic and molecular biology perspectives, demonstrating that SNFH-associated miRNAs are related to the osteoblast differentiation of bone marrow mesenchymal stem cells (BMSCs) involved in lipogenesis, suggesting the potential of miRNAs as biomarkers and therapeutic targets for SNFH [6, 7].

N6-methyladenosine (m6A), a prevalent epigenetic modification, involves the methylation of the nitrogen atom at the 6th position of RNA adenosine [8]. This modification strongly impacts biological processes such as alternative splicing, RNA transport, translation, and degradation. Dysregulation of m6A methylation has been linked to various diseases [9, 10]. The enzymes and proteins associated with RNA m6A methylation can be classified into three groups: m6A transferase, m6A desmethyltransferase, and m6A-binding proteins [11]. The regulatory effects of m6A extend across diverse bodily functions, including germ cell development, embryogenesis, cellular differentiation, metabolism, immune response, and tumorigenesis, as evidenced by numerous studies [12]. Despite growing research on the role of m6A in various diseases, its involvement in SNFH remains underexplored.

Our previous work demonstrated the protective effect of miR27a in SNFH and its potential regulatory role in BMSCs [13]. In bone-related diseases, miR27a was shown to promote osteogenesis by regulating the TGF-β/Smad7 signaling pathway in osteoblasts [14]. miR27a may promote the osteogenic differentiation of bone marrow mesenchymal stem cells in hormonal osteonecrosis of the femoral head by targeting and regulating the PI3K gene [15]. Therefore, miR27a has high potential in osteogenic differentiation. This study aimed to elucidate how miR27a regulates SNFH-derived BMSCs through the m6A-related factor YTHDF2.

## Materials and methods

### Clinical specimens

Bone marrow and serum samples were collected from patients with SNFH and femoral neck fracture (10 patients each) at the Fifth Affiliated Hospital of Xinjiang Medical University from 2019 to 2023. The inclusion criteria for patients with SNFH were as follows: Steinberg stage IV disease according to X-ray or MRI, a history of glucocorticoid use, no history of hip trauma, no history of renal bone disease, and no history of other abnormal bone metabolic disorders, such as ankylosing spondylitis. The inclusion criteria for patients with femoral neck fracture were patients who needed to undergo one or both hip arthroplasty procedures for Garden stage II, III or IV femoral neck fracture and patients who did not have a history of alcohol consumption, cigarette smoking, glucocorticoid use, or other bone metabolic disorders. Information on the clinical samples is shown in Table 1.

**Table 1:** Clinical characteristics of patients

	SNFH (*n* = 10)	Control (*n* = 10)	*P*-value
Age (years)	60.0 ± 3.2	63.0 ± 5.2	0.13
Gender (Male/Female)	6/4	4/6	0.65
BMI (kg/m^2^)	22.86 ± 2.92	22.8 5 ± 3.83	0.99
Total Cholesterol(mmol/L)	6.33 ± 0.52	4.73 ± 0.62	0.001
Glucocorticoid Medication	10 (100%)	2 (20%)	0.001

### Extraction of RNA from clinical BMSC samples

BMSCs from clinical samples (experimental and control groups) were harvested and lysed in 200 µL of TRIzol reagent (Invitrogen) to quantify RNA expression. After incubation, precooled chloroform was added, and RNA was subsequently extracted and purified. The expression levels were then determined in 96-well plates via an enzyme labeling system.

### Modeling of BMSCs for SNFH

Human BMSC progeny were procured from Procell (Wuhan, China), cultivated, and propagated to the third generation. Subsequently, human BMSCs were subjected to methylprednisolone treatment (Pfizer, USA) to establish a model of SNFH BMSCs.

### m6A total RNA quantification

For the quantitative analysis of total m6A RNA in the serum samples, we used the EpiQuik m6A RNA Methylation Kit. Initially, the binding solution was prepared. Subsequently, 80 µL of antibody was added to each well of the 96-well plates, and the plates were incubated in an incubator at 37 °C for 90 min. Next, 50 µL of antibody was added to each well, followed by the addition of a fluorescent solution. The plates were then incubated at room temperature in the dark for 4 min. The absorbance was subsequently measured at 450 nm using an enzyme marker.

### Cell cultures and transfection

Initially, the BMSCs were resuspended in DMEM containing 100 IU/ml penicillin and 100 µg/ml streptomycin. The cells were then incubated in a CO_2_ incubator at 37 °C. YTHDF2, negative control plasmid (NC), siRNA-YTHDF2, and siRNA-NC were obtained from Gemma Pharmaceuticals (Shanghai, China). Transfection of plasmids and siRNAs into BMSCs was carried out using Lipofectamine 2000 (Invitrogen), and the transfection efficiency was assessed using Western blotting (WB) and quantitative real-time polymerase chain reaction (qRT‒PCR).

### Quantitative real-time PCR

The primers were designed using Primer Premier 5.0 software, with the U6 gene serving as the internal reference. The primer sequences were sent to Shanghai Biological Company for synthesis, and the synthetic sequences are presented in Table 1. The concentration and purity of the extracted RNA were assessed through spectrophotometry. Reverse transcription of cDNA was carried out following the instructions of the reverse transcription kit, and the reverse transcription process utilized Power SYBR Green (TaKaRa, China). The results were analyzed using a real-time fluorescence quantitative PCR instrument. Thresholds and baselines were adjusted based on the internal reference data to determine the Ct value for each group of samples. The validity of the Ct value was confirmed through melting curve analysis. Finally, the data were analyzed using the 2^−ΔΔCT^ method. The primer sequences are shown in Table 2.

**Table 2:** PCR Primer sequence et al. in this Study

Genes	Primer
YTHDF2	F:5′-CGGUCCAUUAAUAACUAUATT-3′ R:5′-UAUAGUUAUUAAUGGACCGTT-3′
miR27a	F:5′-TGCGGTTCACAGTGGCTAAG-3′ R:5′-CTCAACTGGTGTCGTGGA-3′
U6	F:5′-CTCGCTTCGGCAGCACA-3′ R:5′-AACGCTTCACGAATTTGCGT-3′
hsa-RUNX2	F:5′-TGAGCGACGTGAGCCCGGTA-3′ R:5′-CGTGTGGAAGACAGCGGCGT-3′
hsa-ALP	F:5′-CTGGAGTCCTGCTCTGTGTG − 3′ R:5′-GGCACTTCTTGAAAGCCTGC-3′
hsa-OCN	F:5′-CCACCGAGACACCATGAGAG-3′ R:5′-CGCCTGGGTCTCTTCACTAC-3′
hsa-PPARγ	F:5′-CCAGAAGCCTGCATTTCTGC-3′ R:5′-CACGGAGCTGATCCCAAAGT-3′
hsa -C/EBP-α	F:5′-TATAGGCTGGGCTTCCCCTT-3′ R:5′-AGCTTTCTGGTGTGACTCGG-3′

#### Western blot (WB)

Proteins were extracted from BMSCs and bone marrow tissues using RIPA lysis buffer on ice. The primary antibody was diluted with primary antibody diluent (1:1000), and the desired bands were excised based on marker positions, followed by incubation with the corresponding primary antibody. The secondary antibody (1:2000) was diluted with TBST buffer, and the membrane was incubated with the target bands for 2 h at room temperature on a shaker. β-Actin was used as the internal reference protein. Subsequently, the bands were photographed and subjected to quantitative analysis of gray values using ImageJ software.

### Download of microarray data

The SNFH dataset GSE123568 was downloaded from the Gene Expression Omnibus (GEO) database (https://www.ncbi.nlm.nih.gov/geo/*)* [16, 17]. Data cleaning, correction, and normalization were performed using the log2 function in R software (version 4.2.1).

### Expression analysis of differentially expressed genes (DEGs)

The differential analysis of gene expression levels between the disease and normal groups was conducted using the R software “limma” package [18]. Differentially expressed genes (DEGs) were screened based on the following criteria: |log2FC| > 1 and p value < 0.05. For visualization of the differential expression patterns of m6A-regulated genes in the GSE123568 dataset, heatmaps were generated using the “ggplot2” [19] and “pheatmap” packages in R software. ROC curves were constructed for the identified YTHDF2-associated DEGs using the “pROC” package in R software (version 1.16.2) [20].

### GSEA

The median values of the biomarkers were stratified into groups characterized by high and low expression levels. Subsequently, the samples within the high- and low-expression groups were analyzed for discrepancies utilizing the “limma” package. The logFC values obtained were sorted in descending order, followed by the implementation of gene set enrichment analysis (GSEA). The enrichment analysis of GSEA was conducted utilizing the “clusterProfiler” package in R software [21], with a significance threshold set at *P* < 0.05. This analysis aimed to identify common functions and pathways associated with many genes within the set of differentially expressed genes.

### CCK-8 assay

The BMSCs were grouped and individually prepared as single-cell suspensions in 96-well plates. Except for the blank group, each well received 100 µL of cell suspension containing 2 × 10^3^ cells/well. The plates were then placed in a CO_2_ incubator, allowing the cells to adhere to the well walls. At the specified time points, the 96-well plate was removed, and 10 µL of CCK-8 solution was added to each well, followed by incubation for 2 h. Finally, the absorbance values were measured at 490 nm using an enzyme-linked immunoassay.

### TUNEL assay

The TUNEL reaction solution was applied to cover the cells once the grouped BMSCs had adhered to the slide. The DAPI dye storage solution was then diluted 100-fold with PBS to create a working solution of 10 g/ml. A drop of the DAPI working solution was applied to the cell slice, ensuring coverage of all cells. Drops of antifluorescence quenching sealer were added to the cell slides, and coverslips were used to seal them. Finally, the slides were cleaned with deionized water and observed under a microscope.

### Osteogenic induction (OI) and Alizarin Red staining

Different groups of BMSCs were induced using osteogenic induction medium supplemented with 50 mmol/L ascorbic acid, 100 nmol/L dexamethasone, 10 mmol/L β-glycerophosphate, and 15% FBS for 15 days. Following the induction period, the BMSCs were fixed in 4% paraformaldehyde for 20 min and subjected to three washes with PBS. Subsequently, the cells were stained with 4 mg/ml Alizarin Red (pH 8.3) for 30 min, followed by three additional PBS washes. The stained cells were photographed, and optical density (OD) values were measured.

### Lipogenic induction and oil red O staining

Distinct subgroups of BMSCs were cultivated in lipid-forming induction medium supplemented with 10 µg/ml insulin, 1 µmol/L dexamethasone, 0.5 mmol/L MIBMX, and 0.1 mmol/L indomethacin for 15 days. Subsequently, the BMSCs were fixed with paraformaldehyde for 1 h and stained for 30 min in the absence of light using an Oil Red O solution. The cell morphology was then observed and imaged under a microscope.

### Statistical analysis

The data were analyzed utilizing SPSS 27.0 software. Differences between two groups were assessed using t tests, and one-way ANOVA was used to compare differences among multiple groups. For the bioinformatic analysis experiments, statistical methods provided by the R package in the R language were utilized. The experiment was conducted in triplicate, and a P value < 0.05 was considered to indicate statistical significance.

## Results

### Downregulation of miR27a and dysregulated m6A modification via YTHDF2 in SNFH BMSCs

**Fig. 1:**
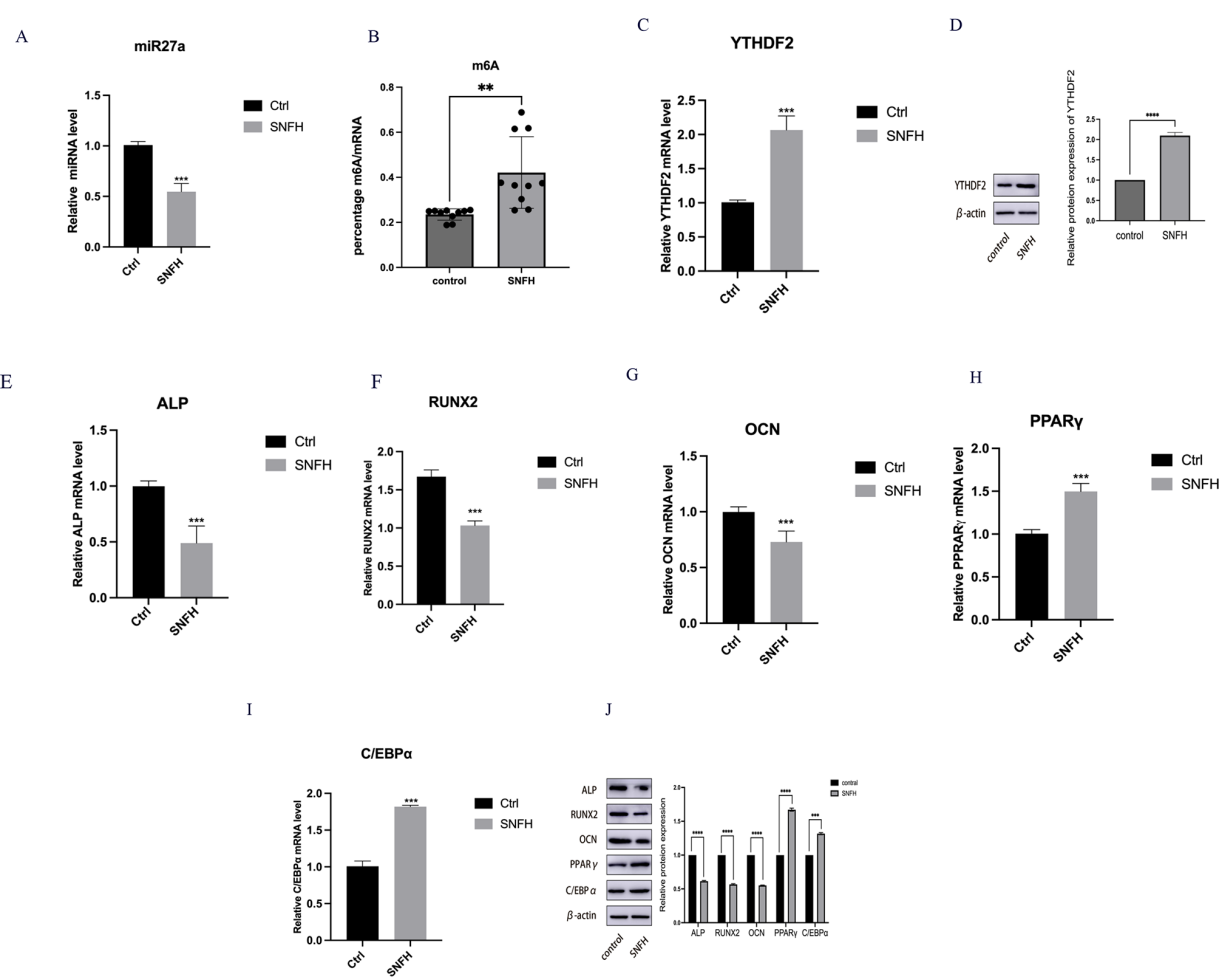
m6A methylation involving miR27a and YTHDF2 is responsible for SNFH. **A**: miR-27a expression in the SNFH group (*n* = 10) and control group (*n* = 10). **B**: Quantification of total m6A-methylated mRNAs in the SNFH and control groups. **C**, **D**: YTHDF2 expression was determined in the SNFH and control groups using WB and qRT‒PCR. **E**-**J**: The expression of osteogenesis-related factors (ALP, RUNX2, and OCN) and lipogenesis-related factors (PPARγ and C/EBPα) was determined in the SNFH group and control group using WB and qRT‒PCR. *P < 0.05, **P < 0.01, ***P < 0.001, ****P < 0.0001

To assess the expression levels of miR27a and YTHDF2 in patients with SNFH, we obtained bone marrow specimens from patients with SNFH in a clinical setting. The analysis revealed lower expression of miR27a in the SNFH group than in the control group (Fig. 1A). Subsequently, the expression of total m6A mRNA was examined in both the experimental and control groups, revealing a significant increase in m6A methylation levels in the SNFH group (Fig. 1B). In the selective assay of the m6A regulator YTHDF2, an increase in its expression was observed in the SNFH group (Fig. 1C and D). Furthermore, the expression of osteogenic factors (ALP, RUNX2, and OCN) and lipogenic factors (PPARγ and C/EBPα) in both the experimental and control groups was assessed using WB and PCR analyses. The results demonstrated a decrease in osteogenic capacity and an increase in lipogenic capacity in the SNFH group (Fig. 1E-J). Taken together, these findings suggest that the occurrence of SNFH is linked to the reduced expression of miR27a and the abnormally high expression of m6A involving YTHDF2.

### miR27a protects BMSCs and negatively regulates YTHDF2

**Fig. 2:**
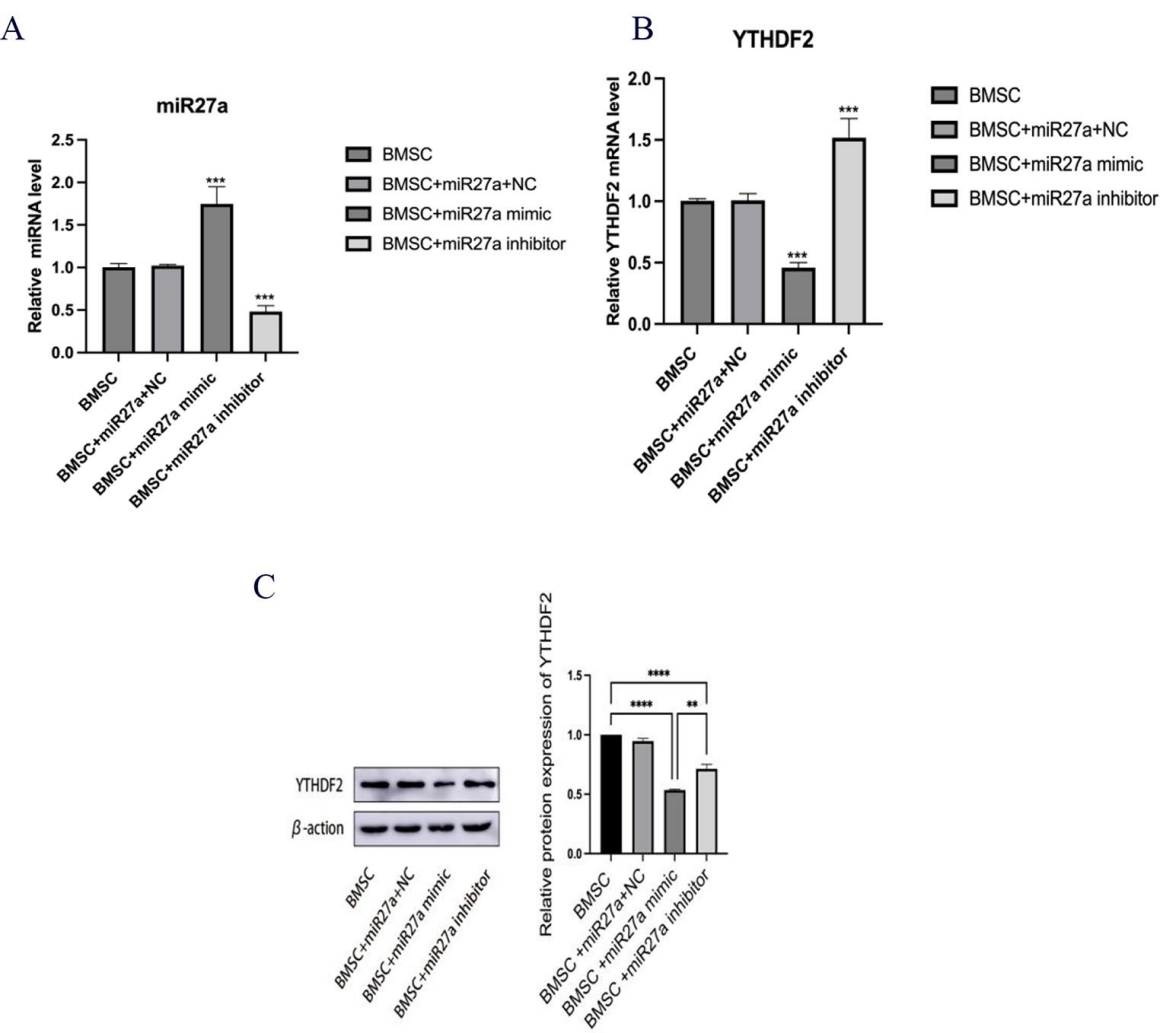
miR27a Overexpression/Inhibition and Its Impact on YTHDF2. **A**: Efficacy of miR27a knockdown and overexpression determined via qRT‒PCR. **B**, **C**: The relationship between miR27a and YTHDF2 expression determined via qRT‒PCR and WB. *P < 0.05, **P < 0.01, ***P < 0.001, ****P < 0.0001. ns, not significant

**Fig. 3:**
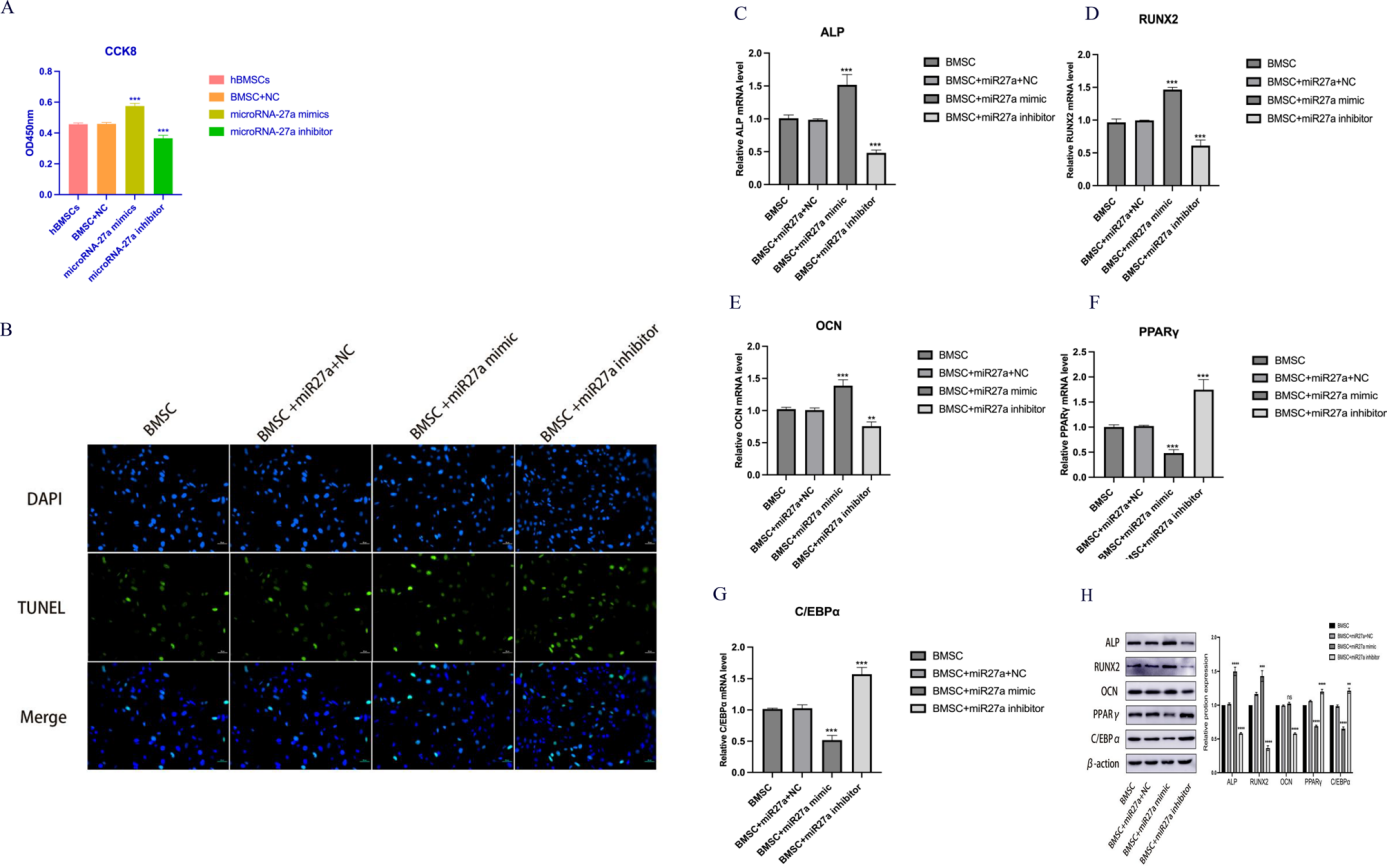
miR27a Overexpression/Inhibition and Its Impact on BMSCs. **A**: The proliferative effect of miR27a on BMSCs was evaluated using a CCK-8 assay. **B**: The apoptotic effect of miR27a on BMSCs was assessed using TUNEL assays, scale bar = 50 μm. **C**-**H**: The expression of osteogenesis-related factors (ALP, RUNX2, and OCN) and lipogenesis-related factors (PPARγ and C/EBPα) in each group was evaluated via WB and qRT‒PCR. *P < 0.05, **P < 0.01, ***P < 0.001, ****P < 0.0001. ns, not significant

To explore the role of miR27a in regulating BMSCs, we performed targeted knockdown and overexpression experiments using a model system. The effective manipulation of miR27a levels was confirmed by qRT‒PCR (Fig. 2A). Notably, miR27a knockdown/overexpression modulated YTHDF2 expression via a negative regulatory relationship, as revealed by WB and qRT‒PCR (Fig. 2B, C). miR27a has a strong gene regulatory effect, and many studies have demonstrated that miR27a regulates the expression of target genes mainly through direct action on these genes. Therefore, we predict that miR27a directly targets YTHDF2 to negatively regulate its expression. Using CCK-8 and TUNEL assays, we demonstrated that miR27a has a protective effect on BMSCs by promoting cell proliferation and division while inhibiting apoptosis (Fig. 3A, B). Further analysis of lipogenic and osteogenic markers in each group confirmed that miR27a promoted BMSC osteogenic differentiation and suppressed BMSC lipogenic potential (Fig. 3C-H).

### The importance of YTHDF2 in SNFH

**Fig. 4:**
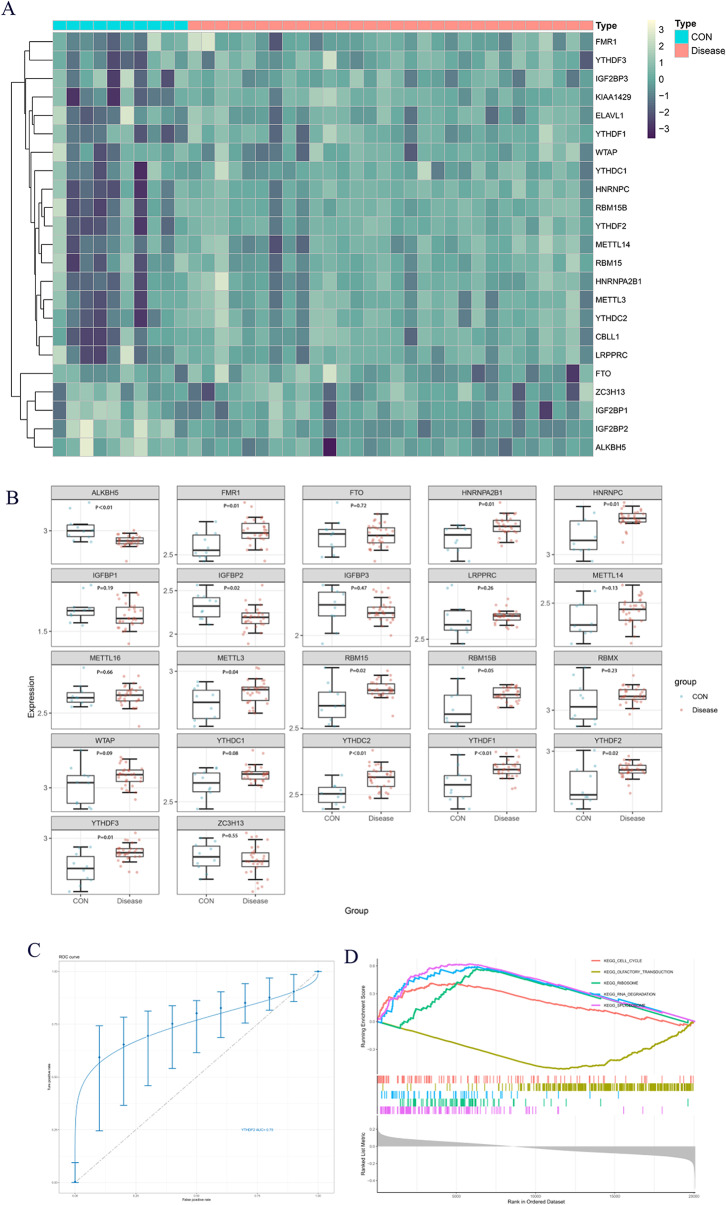
The role of YTHDF2 in SNFH. **A**: Heatmap representing differentially expressed m6A-related genes in the SNFH gene database GSE123568. The X-axis shows the gene set of the experimental and control samples, and the Y-axis is the set of m6A-related genes, where the color shading represents the magnitude of differential expression. **B**: Statistical analysis of m6A-related genes using the Wilcoxon signed-rank test (*P* < 0.05, significant). **C**: Operating characteristic curve for YTHDF2 in GSE123568. **D**: GSEA enrichment analysis for YTHDF2 as a single gene in SNFH. The Y-axis shows the ES score, where a positive ES indicates that a functional gene set is enriched in the front of the sorted sequence, and a negative ES indicates that a functional gene set is enriched in the back of the sorted sequence. In general, four pathways were promoted, and one pathway was inhibited

To explore the role of m6A modification in SNFH, we conducted bioinformatics analysis on the GEO dataset GSE123567. A heatmap was generated to visualize m6A-associated regulators, which indicated their increased expression in patients with SNFH (Fig. 4A). A comparison of m6A-related regulators between the disease and control groups revealed significant differences, with YTHDF2 exhibiting statistical significance at *P* < 0.05 (Fig. 4B). To validate the clinical utility of YTHDF2, we conducted a receiver operating characteristic (ROC) curve analysis, yielding an area under the curve (AUC) value of 0.75. Thus, YTHDF2 can be considered a potential biomarker for SNFH (Fig. 4C). Gene set enrichment analysis (GSEA) of YTHDF2 indicated its involvement in enriched pathways such as “CELL_CYCLE”, “OLFACTORY_TRANSDUCTION”, “RIBOSOME”, and “RNA_DEGRADATION”, while showing the inhibition of the “OLFACTORY_TRANSDUCTION” pathway in SNFH (Fig. 4D). Therefore, the upregulation of YTHDF2 plays a crucial role in the pathogenesis of SNFH.

### Knockdown of YTHDF2 promotes the osteoblast differentiation of BMSCs

**Fig. 5:**
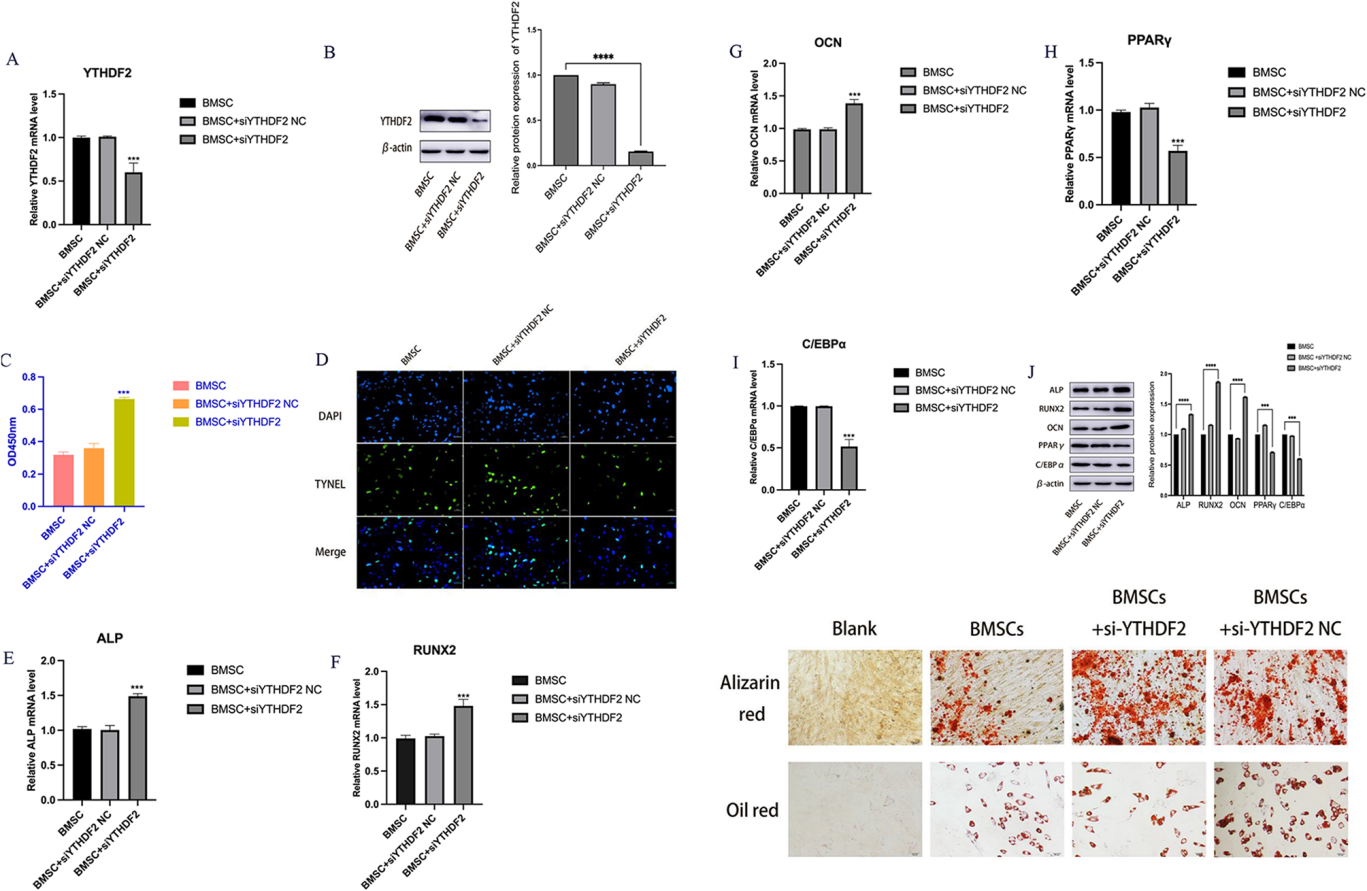
Increased osteogenesis of BMSCs by silencing YTHDF2 in the SNFH group. **A**, **B**: The efficiency of YTHDF2 knockdown was determined by qRT‒PCR and WB. **C**: The proliferative effects of YTHDF2 silencing on BMSCs were evaluated using a CCK-8 assay. **D**: The apoptotic effects of YTHDF2 silencing on BMSCs were assessed by TUNEL staining (scale bar = 50 μm). **E**-**J**: The expression levels of osteogenesis-related factors (ALP, RUNX2, and OCN) and lipogenesis-related factors (PPARγ and C/EBPα) were determined by Western blotting and qRT‒PCR. K: Alizarin red staining after osteogenic induction. Oil red O staining after lipogenic induction (scale bar = 20 μm). *P < 0.05, **P < 0.01, ***P < 0.001, ****P < 0.0001

To investigate the impact of YTHDF2 silencing, we downregulated its expression (Fig. 5A, B). This intervention was observed to increased the proliferation and inhibit the apoptosis of BMSCs (Fig. 5C, D), indicating that decreased YTHDF2 expression promotes the survival of BMSCs in patients with SNFH. Notably, the silencing of YTHDF2 promoted osteogenic differentiation and diminished lipogenic differentiation in BMSCs (Fig. 5E-J), consistent with our earlier identification of the miR27a-mediated negative regulation of YTHDF2. Furthermore, the knockdown of YTHDF2 promoted the osteogenic differentiation of BMSCs, as further validated through Oil Red O staining and Alizarin Red staining (Fig. 5K). In summary, these findings suggest that the reduced expression of YTHDF2 plays a pivotal role in miR27a-mediated BMSC differentiation.

## Discussion

With the widespread use of hormonal drugs, the incidence of SNFH has been increasing over the past few years. Although SNFH is a common condition in orthopedics, there is currently no effective treatment for this condition [22]. Therefore, it has become particularly urgent and important to clarify the pathogenesis of SNFH [23]. With increasing research into the mechanism of SNFH, the fate of BMSCs has been found to play an important role in the development of disease [24]. Under orthopedic conditions, the osteogenic differentiation of BMSCs toward lipid differentiation directly impacts the course of the disease. However, the proliferation of BMSCs and their differentiation direction are influenced by various factors. Accordingly, we sought to identify the key regulators of BMSC differentiation.

Current evidence suggests that miR27a plays an important regulatory role in gene expression and is involved in gene modification at the molecular level in various disease cells. This molecule can reportedly regulate the expression of multiple genes and plays a crucial role in the development of diseases [25]. Numerous studies have shown that miR27a plays a critical role in various diseases, including hyperlipidemia [26], breast cancer [27], and rectal cancer [28]. miR27a may utilized as a potential biomarker for these diseases. In the context of orthopedic diseases, studies have demonstrated that miR27a expression is closely associated with rheumatoid arthritis [29]. Furthermore, high miR27a expression can delay bone loss and counteract bone loss caused by radiation and aging [30]. Interestingly, Hsu et al. [31] reported that miR27a can inhibit osteoclasts by affecting the Squstm1/p62 signaling pathway, which may have a preventive effect on osteoporosis. The present study revealed that reduced osteogenic levels in clinical SNFH samples were associated with low miR27a expression. Decreases in BMSC survival and osteogenic capacity were observed after miR27a knockdown. Therefore, our findings are consistent with previous studies demonstrating the protective effect of miR27a on orthopedic diseases, particularly in relation to BMSC survival.

m6A methylation is the most prevalent and abundant RNA modification, and it modifies a wide range of RNAs, including mRNAs, rRNAs, tRNAs, and miRNAs [32]. In degenerative disc disease, osteoporosis, osteosarcoma, and osteoarthritis, noncoding RNAs play an important role in these diseases by interacting with m6A methylation-associated factors to regulate various biological processes [33]. Many studies have shown that m6A can act on target RNAs to trigger relevant pathological changes, where m6A serves as an upstream factor and the relevant RNA acts as a downstream signaling molecule. For example, in cervical cancer, m6A methylation was shown to alter circRNA modifications, thereby increasing the risk of cervical cancer metastasis^9^. Liu et al. [34] discovered that methylation of noncoding RNAs due to m6A methylation in breast cancer can be utilized to predict relevant therapeutic drug targets and target sites for RNA interactions. Although the regulatory influence of N6-methyladenosine (m6A) on RNA has been extensively documented, microRNAs (miRNAs) offer an additional layer of post-transcriptional control. These noncoding RNA molecules bind to specific target sequences in messenger RNAs (mRNAs), resulting in either decreased translation or mRNA degradation and ultimately regulating gene expression [35].

In the present study, we conducted experiments to demonstrate the role of miR27a in regulating the expression of YTHDF2, a key regulator of m6A methylation, and its impact on the lipogenic/osteogenic differentiation of BMSCs from individuals with SNFH. By specifically knocking down and overexpressing miR27a, we observed that the overexpression of miR27a led to a decrease in YTHDF2 expression, while its knockdown resulted in an increase in YTHDF2 expression. These results indicate that miR27a regulates the expression of YTHDF2, which plays a crucial role in the regulation of lipogenic/osteogenic differentiation in BMSCs from patients with SNFH. Many studies have confirmed that miR27a mainly targets downstream genes through direct targeting; therefore, we predicted that miR27a regulates the expression of YTHDF2 through direct targeting. In conclusion, we predicted that miR27a targeting of YTHDF2-mediated m6A methylation has an important effect on the regulation of lipogenic/osteogenic differentiation in BMSCs from patients with SNFH.

Proteins with the YTF domain act as “readers” in m6A methylation, influencing target mRNA splicing, nuclear translocation, stability, translation, and RNA decay by recognizing and translating m6A sites on various transcripts [36]. The primary function of YTHDF1 is to regulate the translation of modified genes, while YTHDF2 mainly contributes to the degradation of target genes. YTHDF3 collaborates with the former two proteins to either accelerate translation or degradation of the target transcriptome [37]. YTHDF2 stabilizes the transcription of maintenance protein 2 (MCM2) and maintenance protein 5 (MCM5) in microchromosomes, promoting hepatocellular carcinoma progression in hepatitis B virus-infected individuals [38]. In NK cells, YTHDF2 promotes the cellular functions of NK cells and creates the necessary conditions for NK cell survival and proliferation through the formation of a STAT5-YTHDF2 positive feedback loop [39]. Numerous studies have confirmed the involvement of YTHDF2 in the development and progression of various diseases, including glioblastoma [40], liver cancer [41], and malignant glioblastoma [42], highlighting that YTHDF2 plays a crucial role in various diseases. Using bioinformatics research methods, we identified YTHDF2 as a differentially expressed gene in SNFH with clinical diagnostic value. GSEA revealed that YTHDF2 primarily regulates BMSCs in SNFH by promoting cell cycle pathways. Herein, we found that BMSCs exhibited increased proliferative and osteogenic capacities following YTHDF2 silencing, indicating that high YTHDF2 expression promotes the occurrence and development of SNFH.

Currently, the alteration of microcirculation in the femoral head is an important cause of pathological changes and disease progression in patients with SNFH and has been widely recognized by the academic community. Research has revealed that there are many reasons for the deterioration of microcirculation in the femoral head, such as infiltration of immune cells [43] and abnormal deposition of lipids [44]. m6A methylation is strongly associated with the infiltration of immune cells in patients with SNFH and can reduce lipid deposition through the expression of m6A-related genes to improve microcirculation in the femoral head. In the present study, by investigating the effect of low expression of YTHDF2 on reducing the lipogenic differentiation of BMSCs, we expected that a reduction in femoral head lipid deposition would improve the microcirculation. However, further experiments are needed to provide more sufficient evidence for this claim.

At the cellular level, osteoblast aging and disruption of metabolic homeostasis are characteristic pathological changes in SNFH. Many studies have shown that m6A is involved in the regulation of aging in a wide range of cells; for example, the demethylase FTO can delay ovarian aging by reducing the level of methylation in granulosa cells [45]. The regulation of m6A methylation also plays an important role in orthopedic-related diseases, such as osteoporosis and lumbar disc degeneration [46]. Unfortunately, the effect of m6A methylation on osteoblast aging was not further investigated in this study, but we hope to alleviate or treat SNFH by promoting the osteogenic differentiation of MSCs and bone homeostasis in the femoral head.

In summary, we provided compelling evidence that miR27a exerts a protective effect on SNFH by negatively regulating YTHDF2 expression, leading to a reduction in m6A methylation in BMSCs. Currently, the clinical diagnosis of hormonal osteonecrosis of the femoral head is mainly based on imaging to assess the blood supply of the femoral head to predict the risk of hormonal osteonecrosis of the femoral head. However, as early imaging changes in hormonal osteonecrosis of the femoral head are not obvious, the clinical diagnosis often misses the optimal treatment period. Therefore, the expression of miR27a and other related genes can be determined to increase the clinical evidence for the early diagnosis of hormonal osteonecrosis of the femoral head. These findings offer novel insights into the early diagnosis and treatment of SNFH.

### Electronic supplementary material

Below is the link to the electronic supplementary material.

Supplementary Material 1

## Data Availability

The datasets used and/or analysed during the current study available from the corresponding author on reasonable request.
