# Supplementary material for: YTHDF2-Mediated m6A methylation inhibition by miR27a as a protective mechanism against hormonal osteonecrosis in BMSCs
Source: BMC Musculoskelet Disord. 2024 May 6;25:359. doi: 10.1186/s12891-024-07481-3 (PMC11071322; doi:10.1186/s12891-024-07481-3)

Figure 1 YTHDF2 Figure 1 ALP


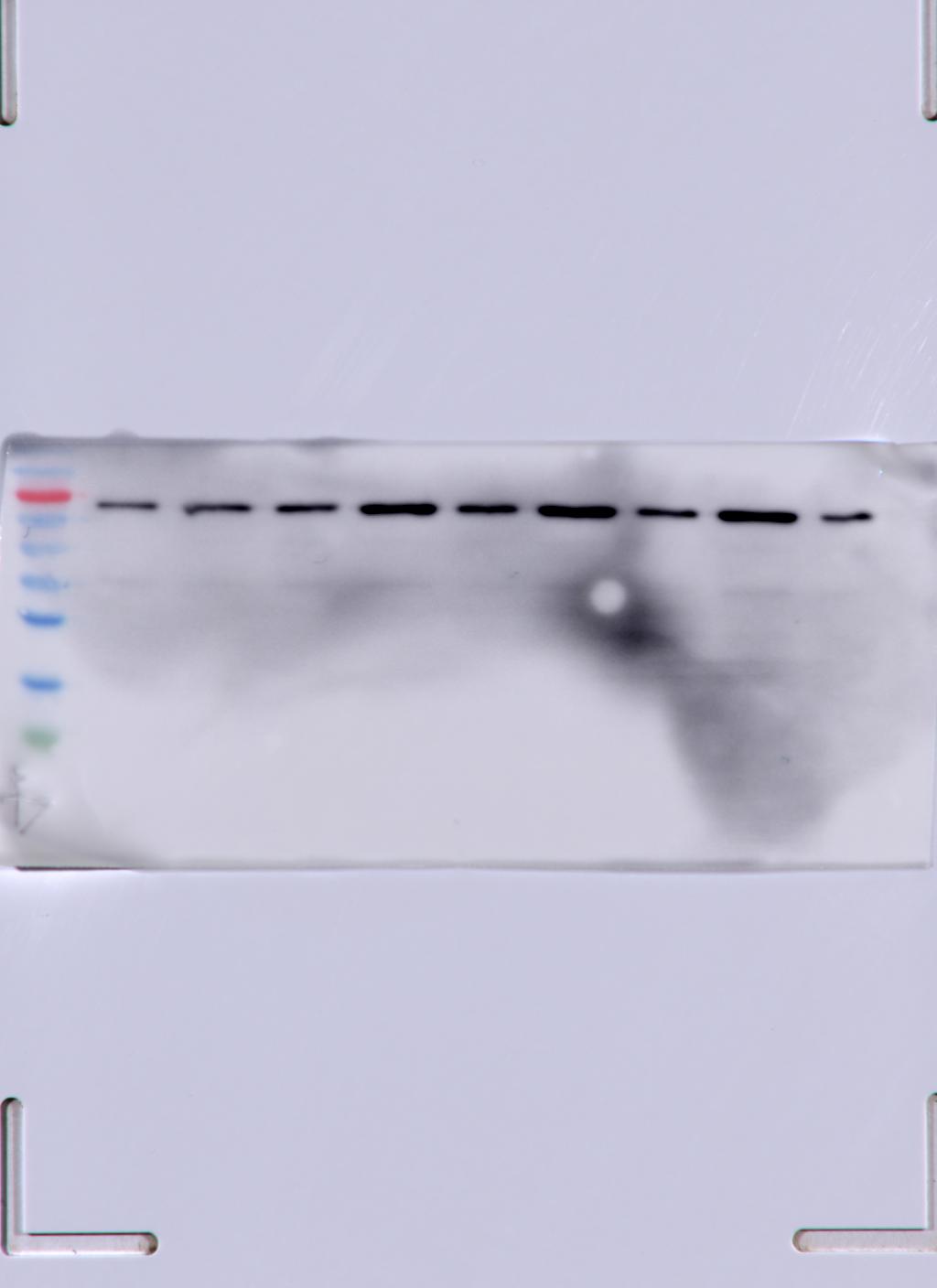

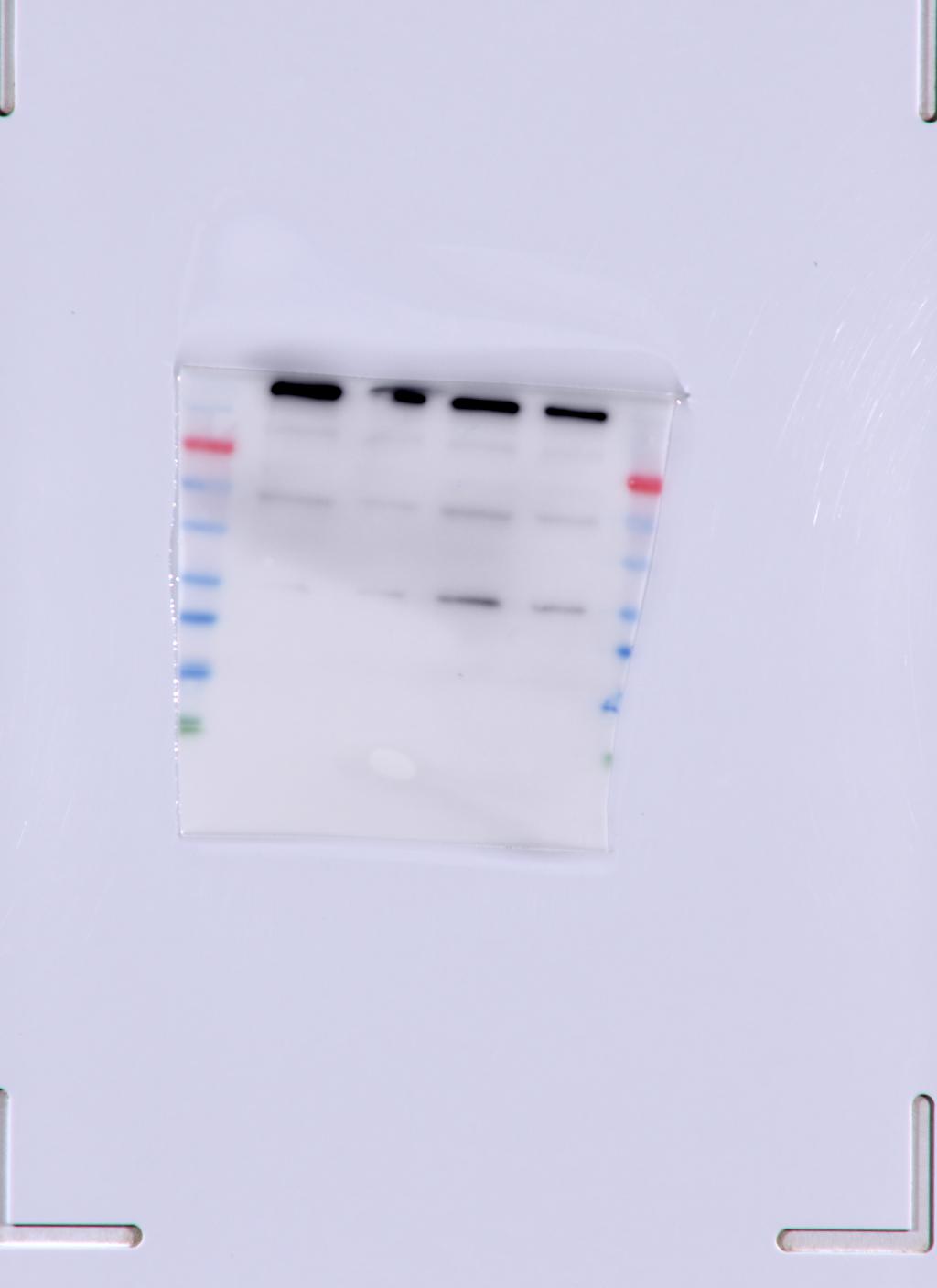


Figure 1 RUNX2 Figure 1 OCN


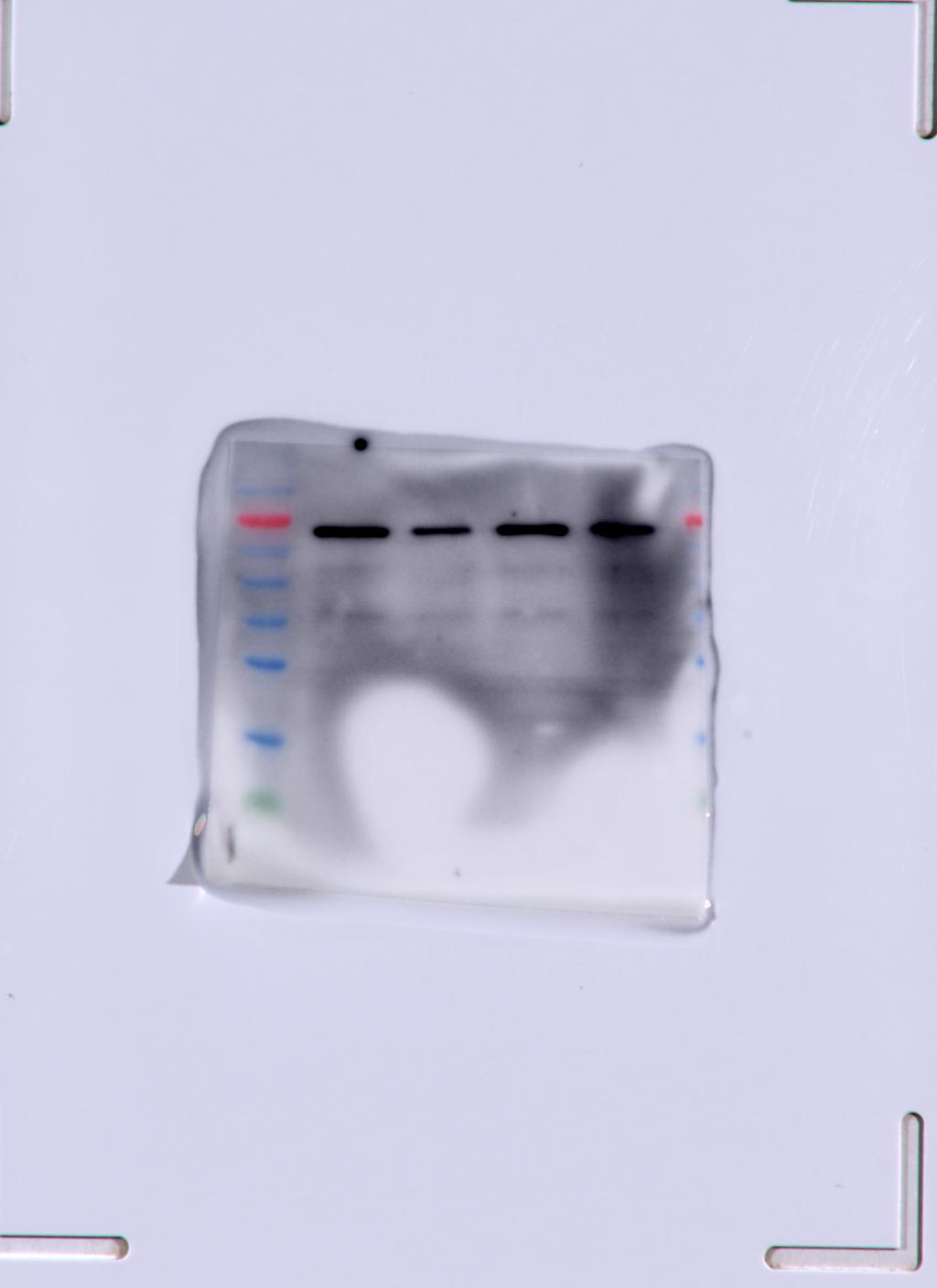

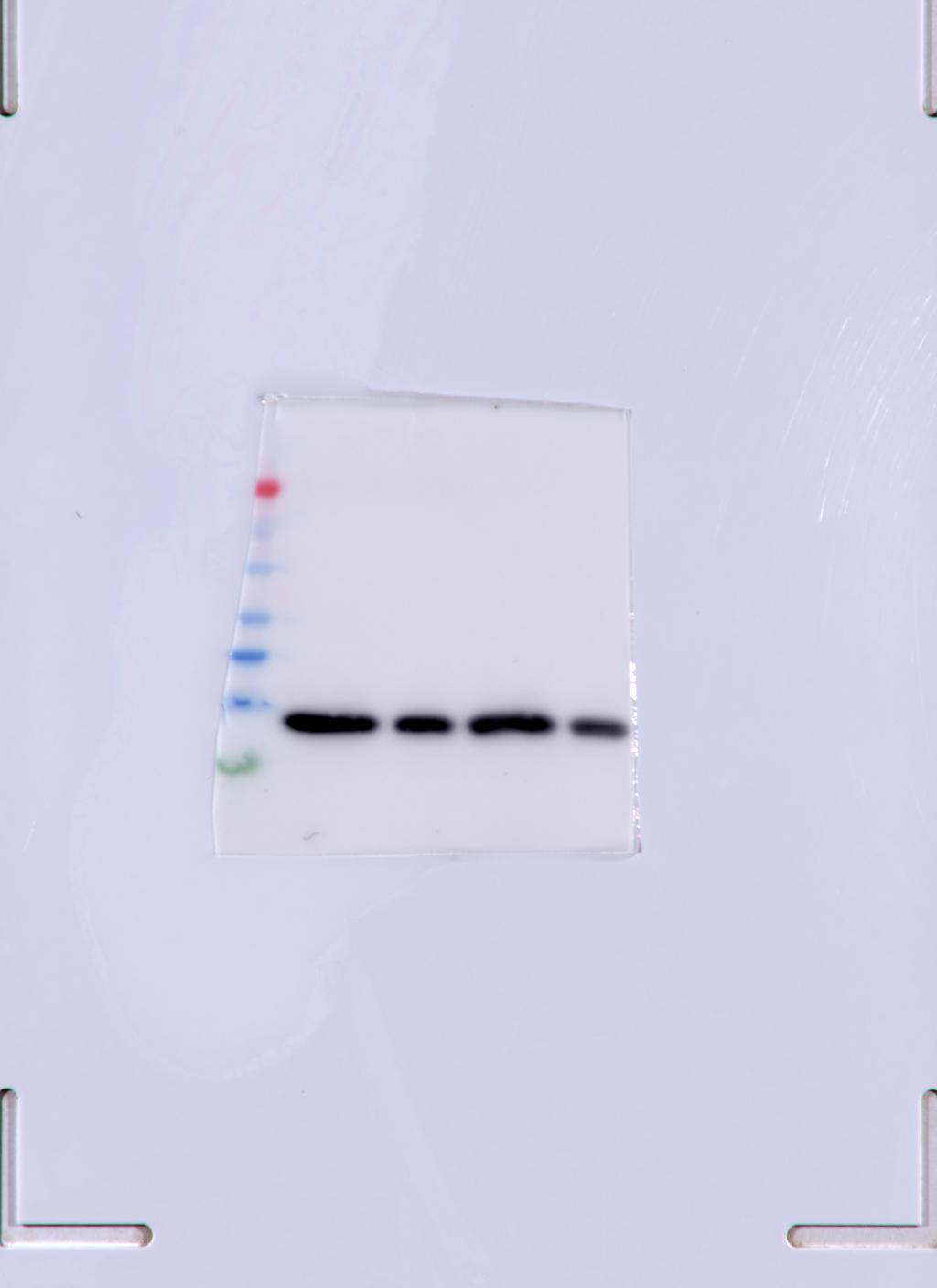


Figure 1 PPARγ Figure 1 C/EBPα


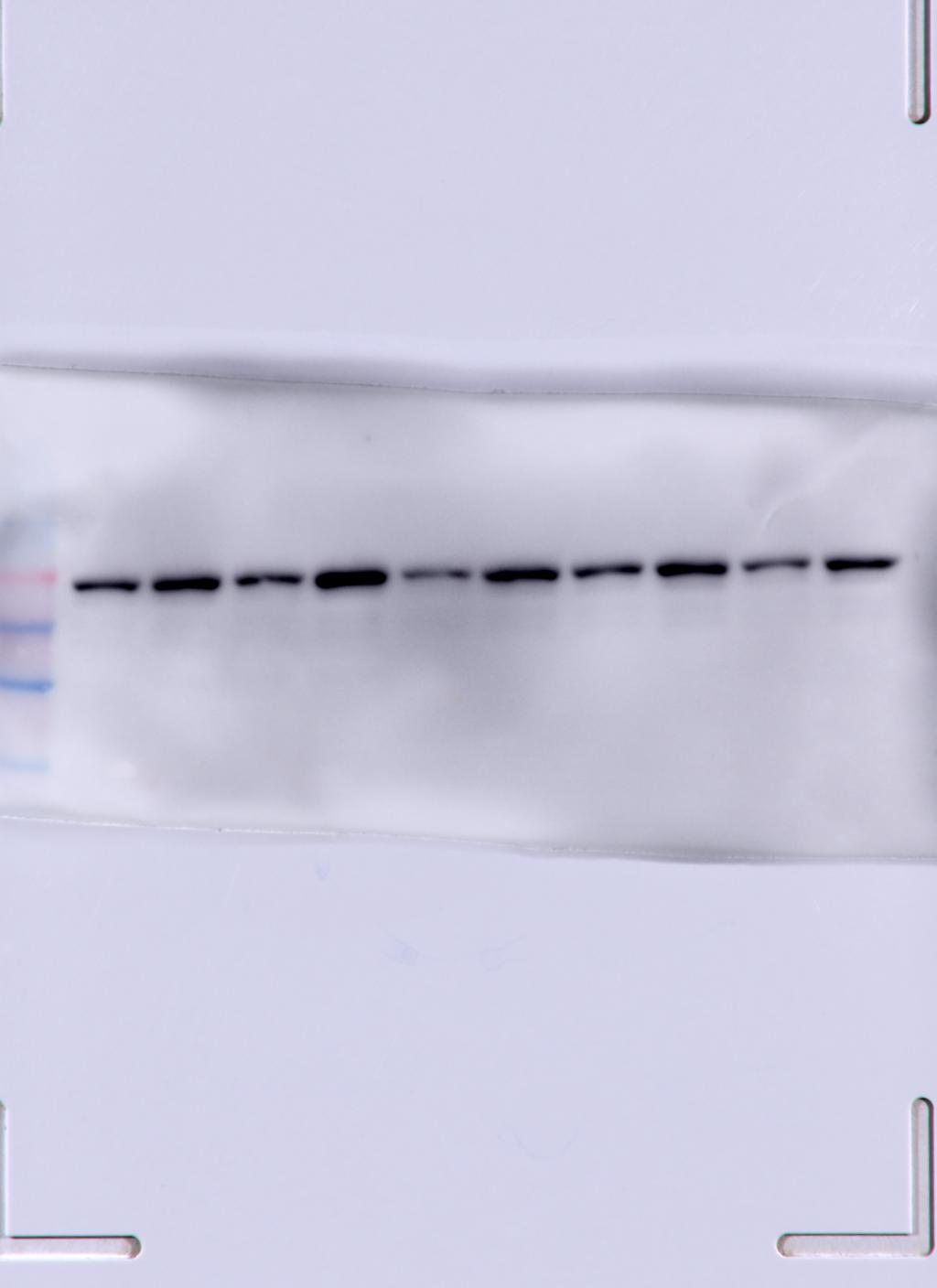

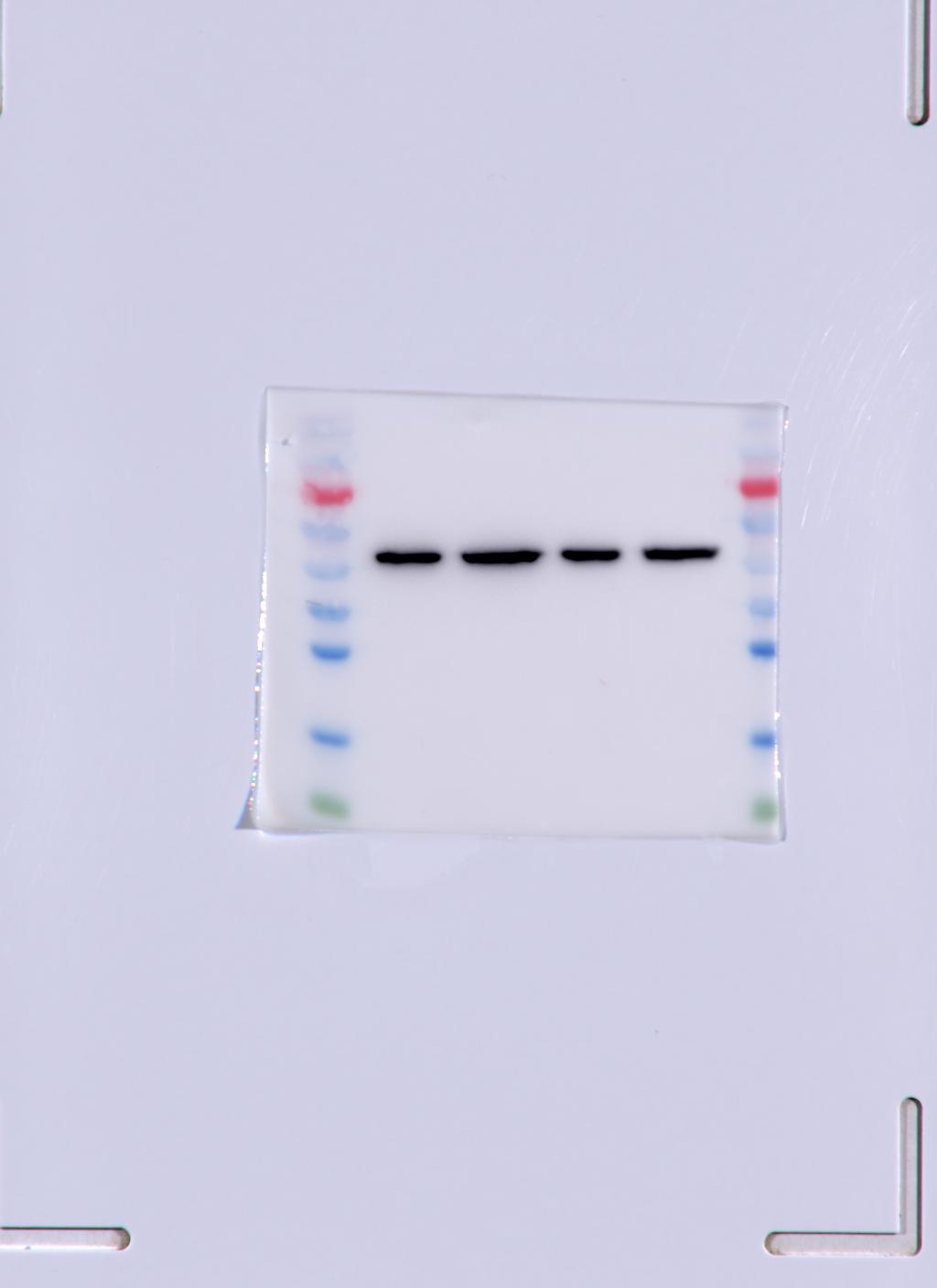


Figure 1 β-action


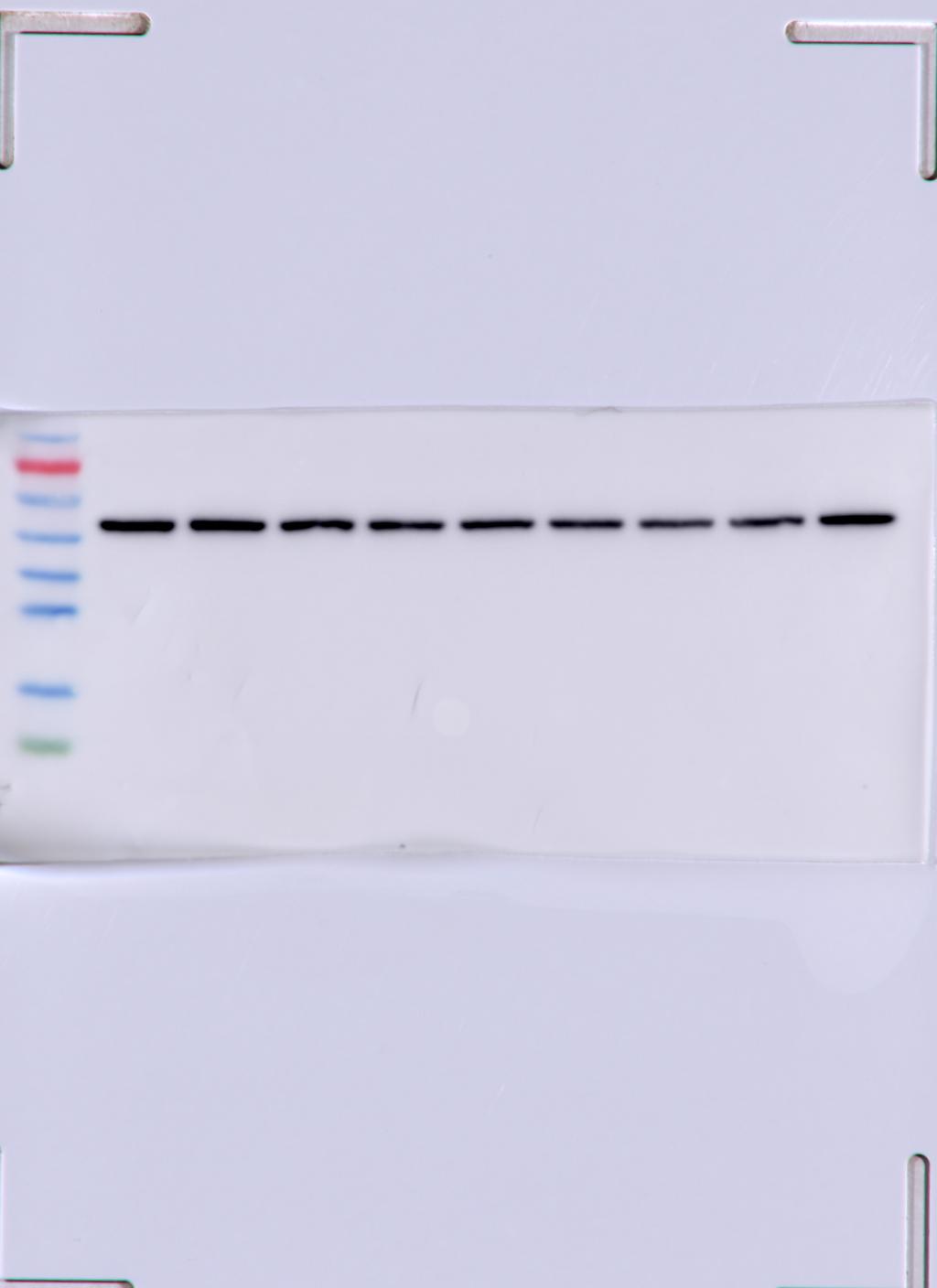


Figure 2 YTHDF2 Figure 3 ALP


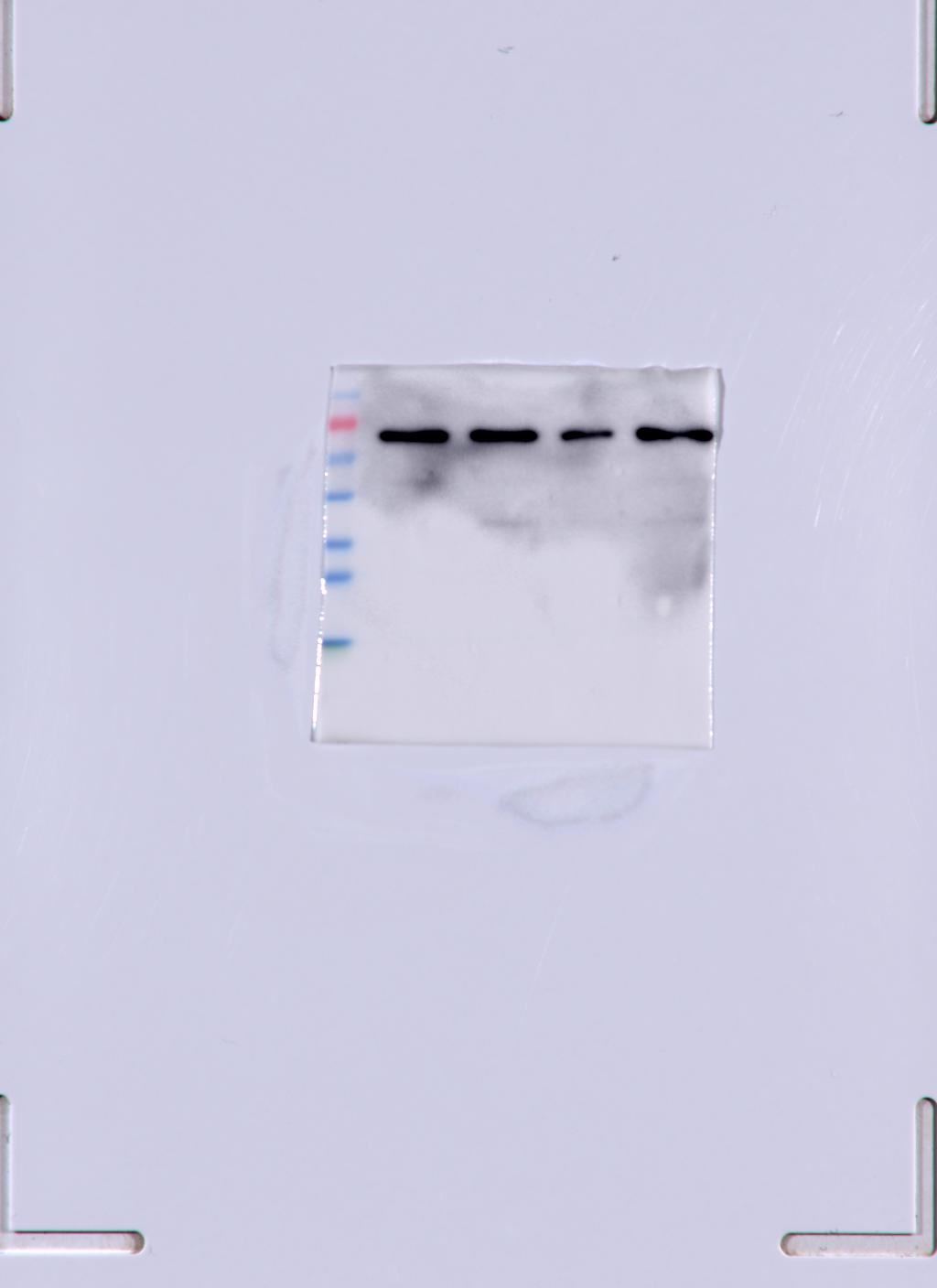

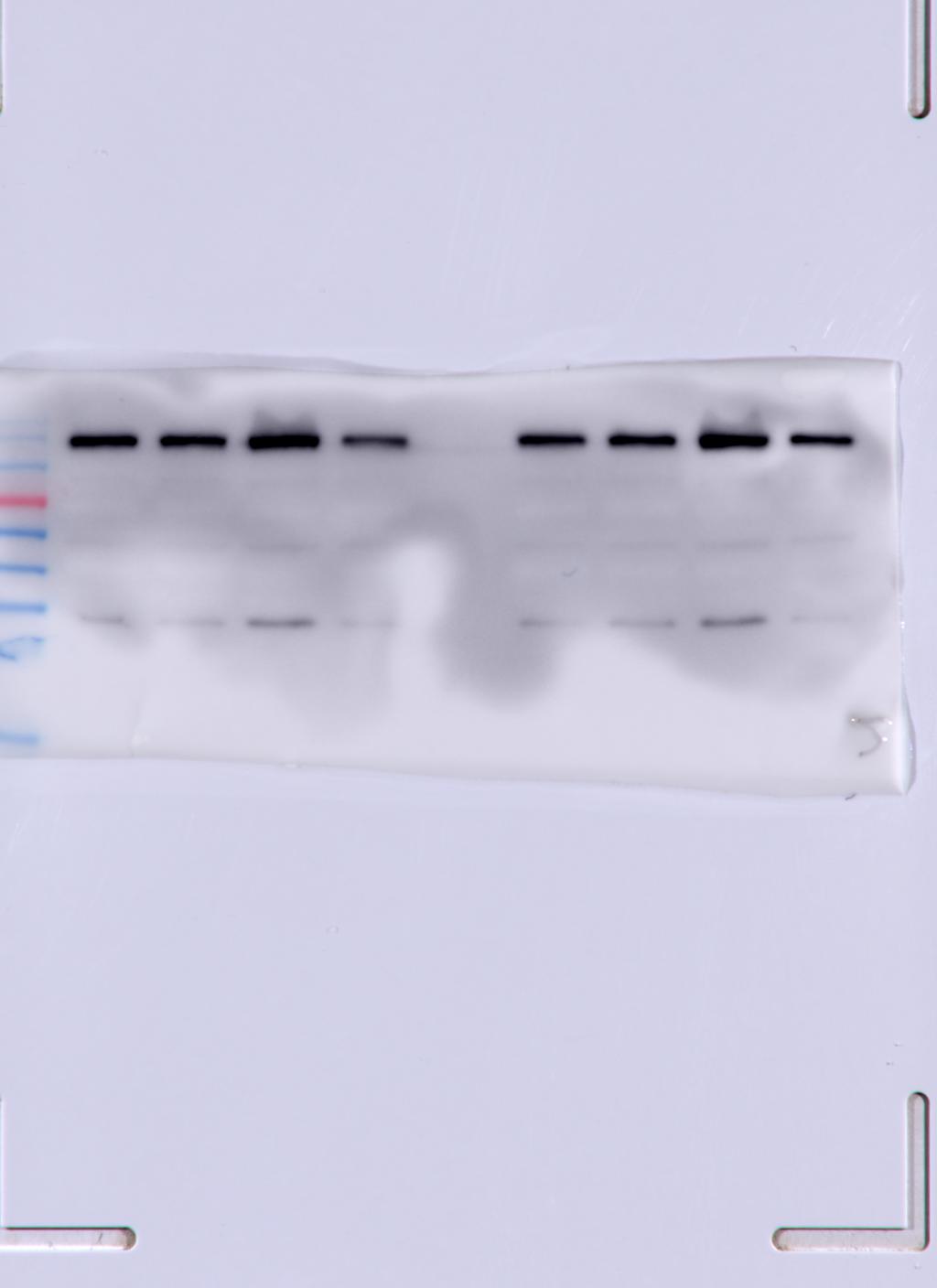


Figure 3 RUNX2 Figure 3 OCN


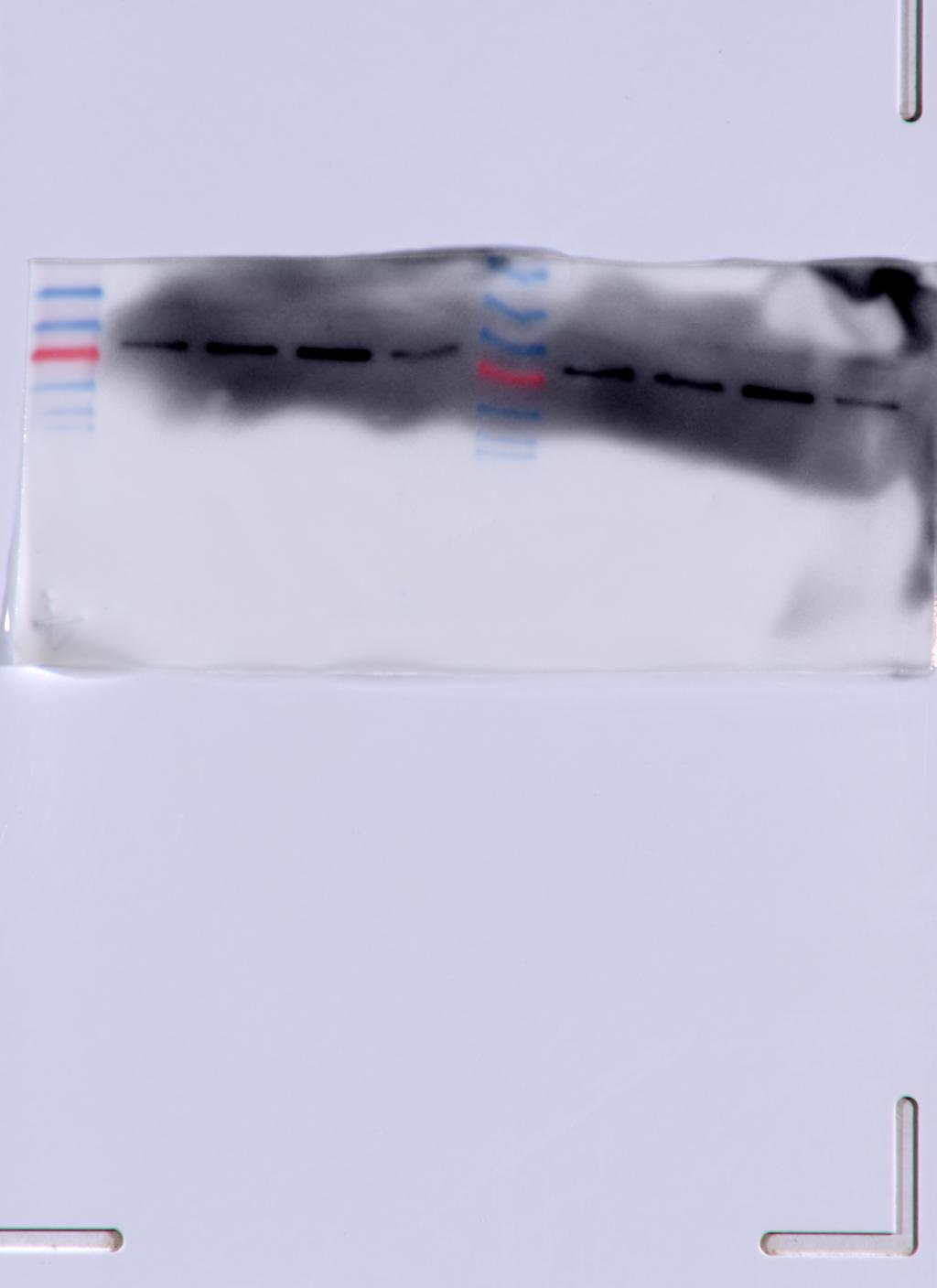

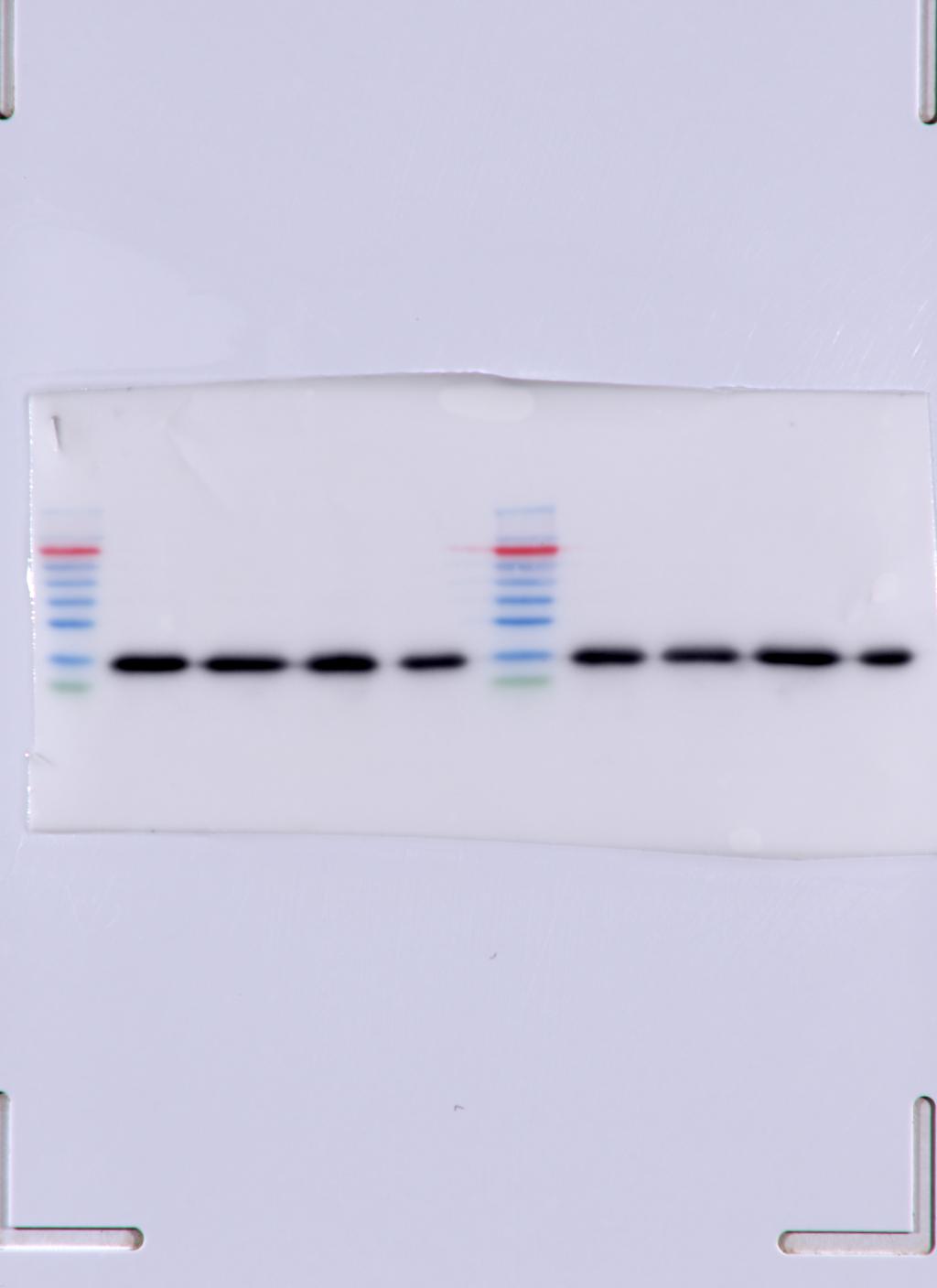


Figure 3 PPARγ Figure 3 C/EBPα


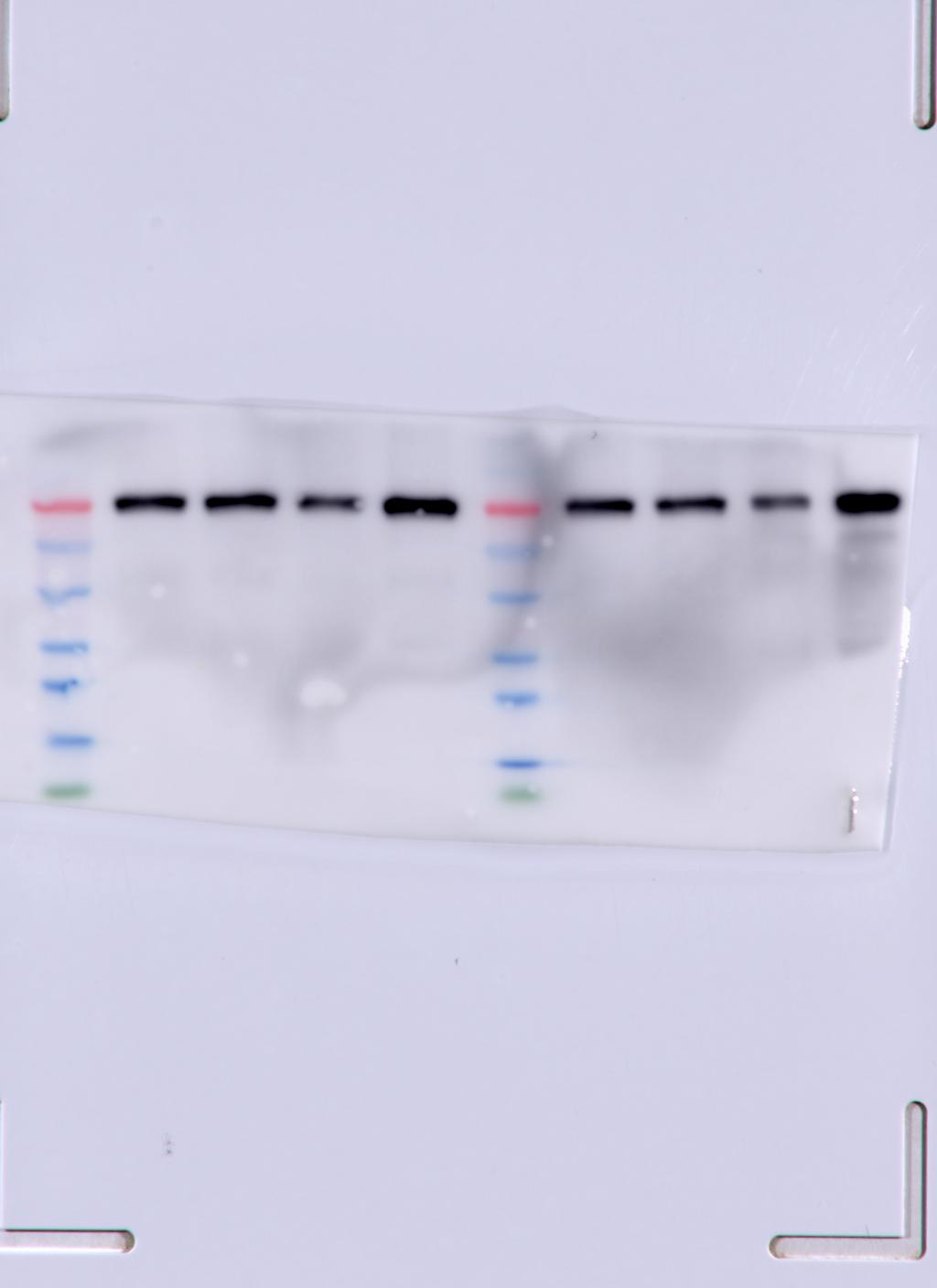

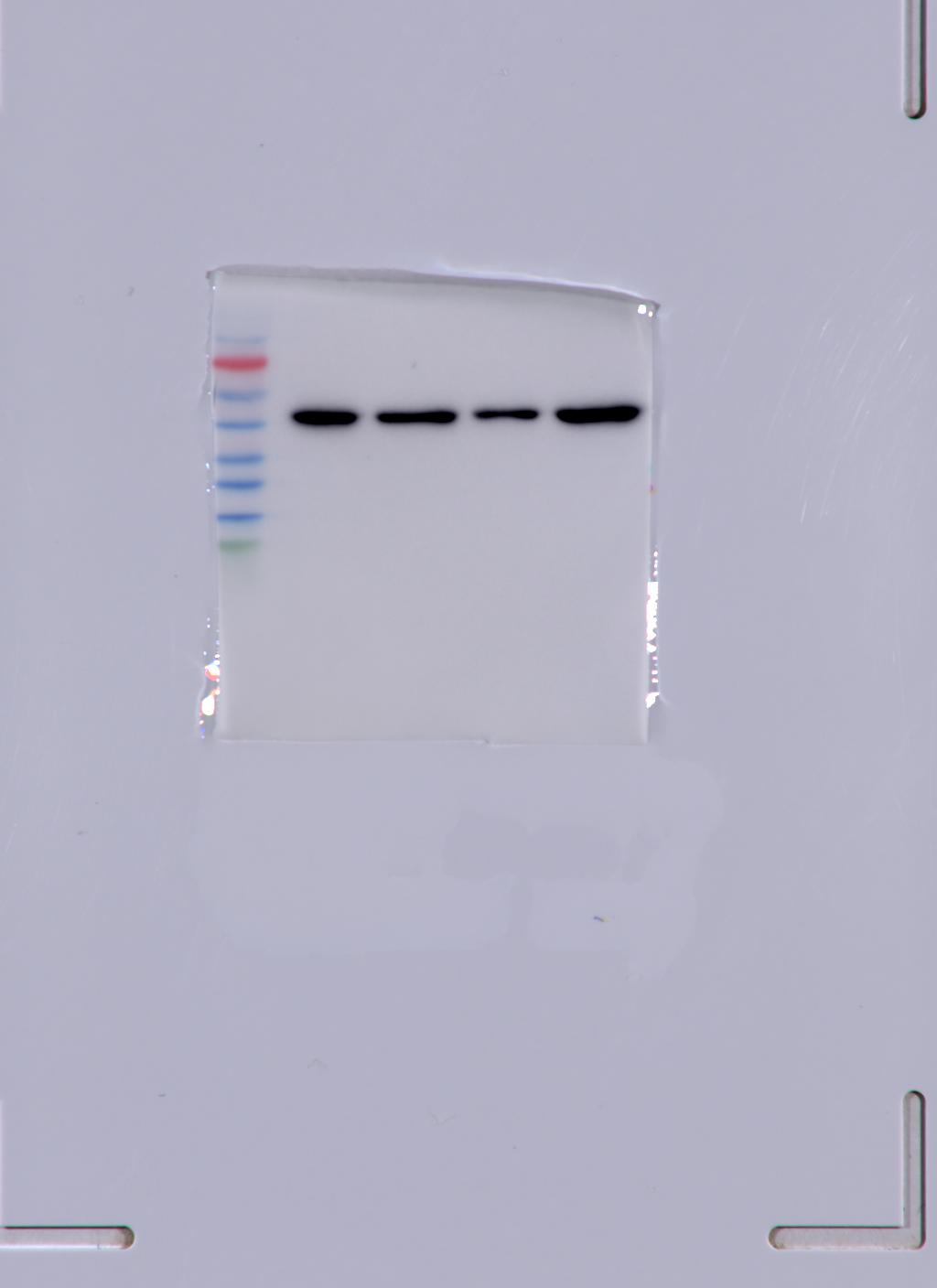


Figure 2 and 3 β-action


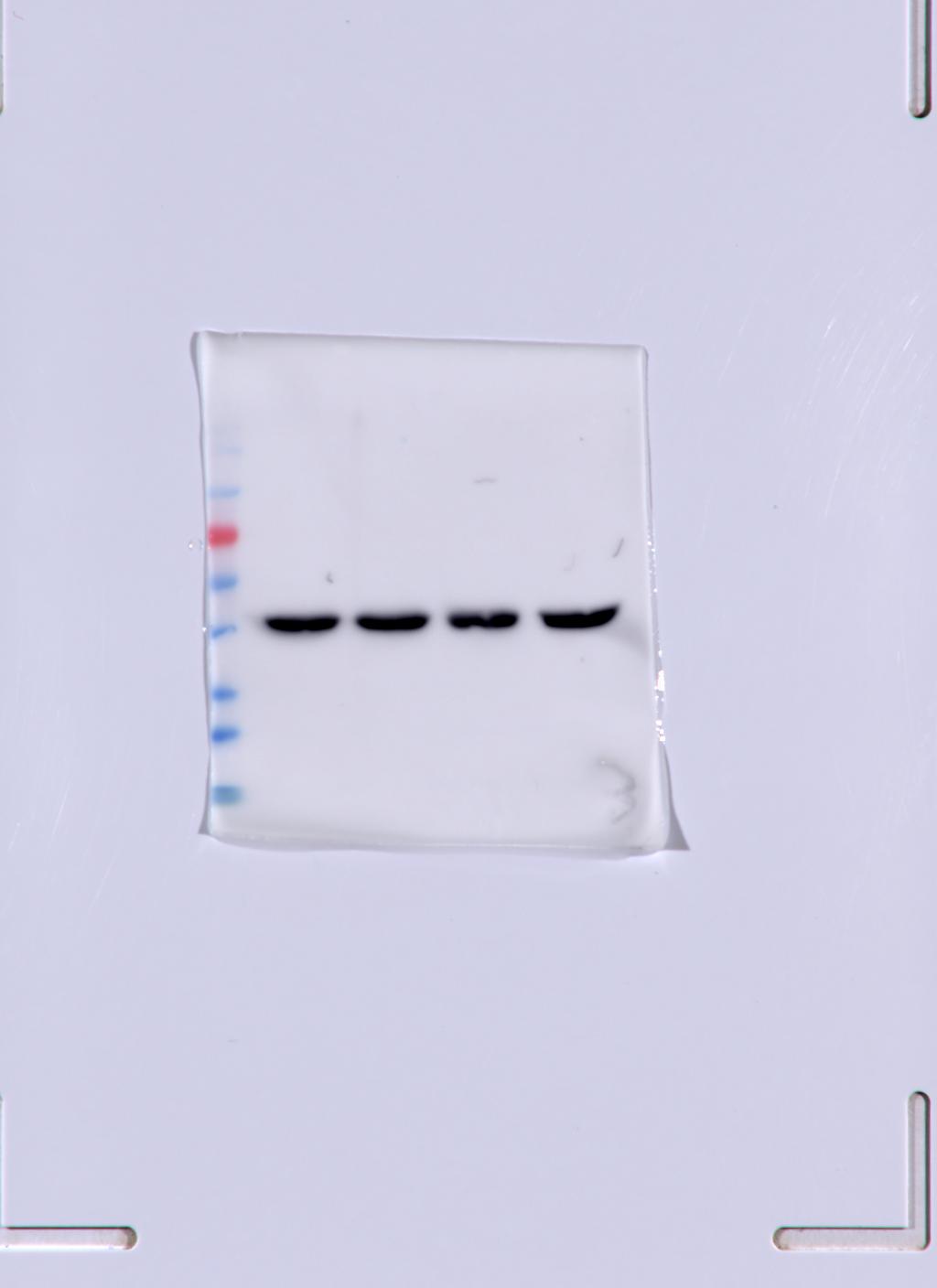


Figure 5 YTHDF2 Figure 5 ALP


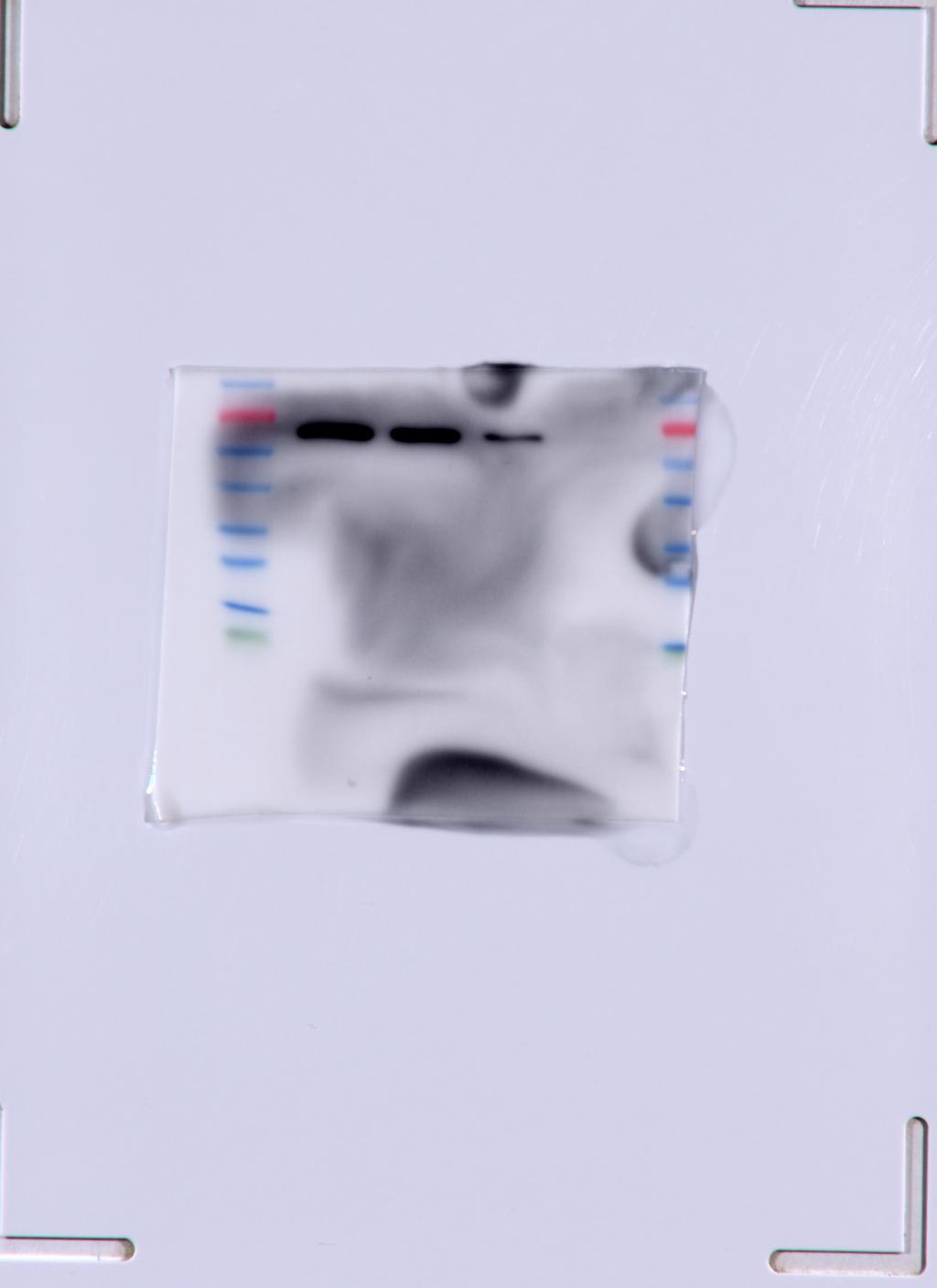

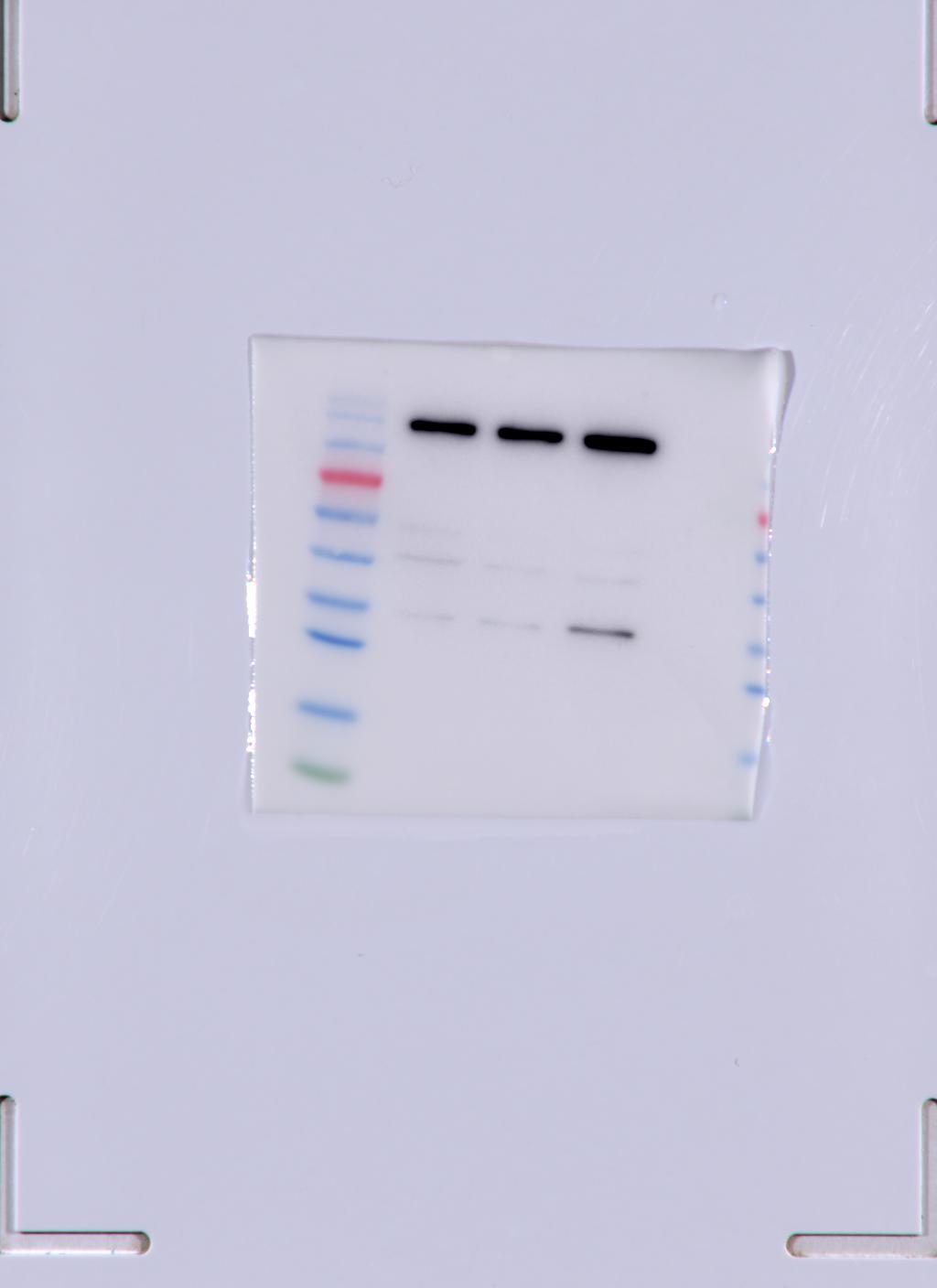


Figure 5 RUNX2 Figure 5 OCN


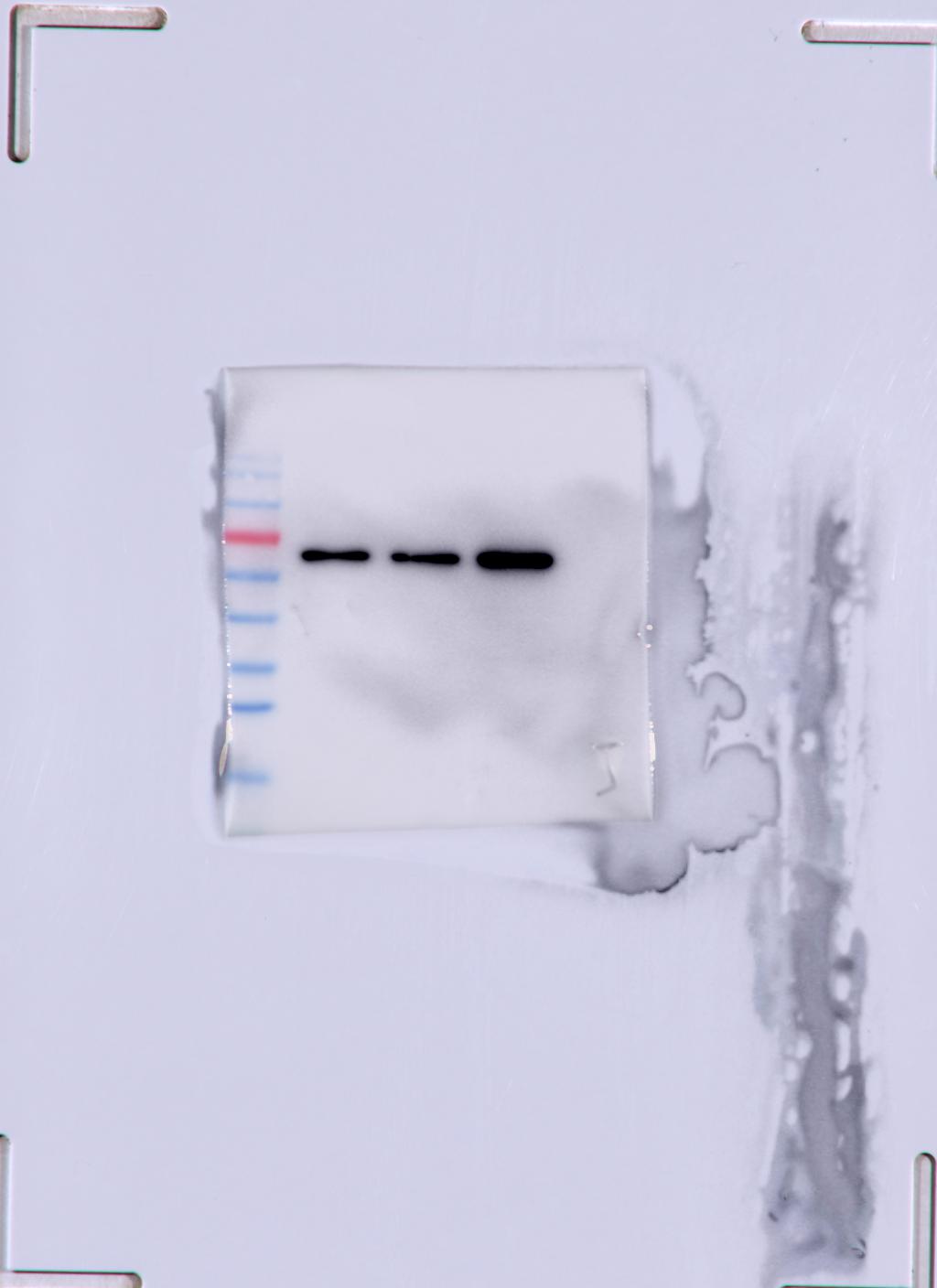

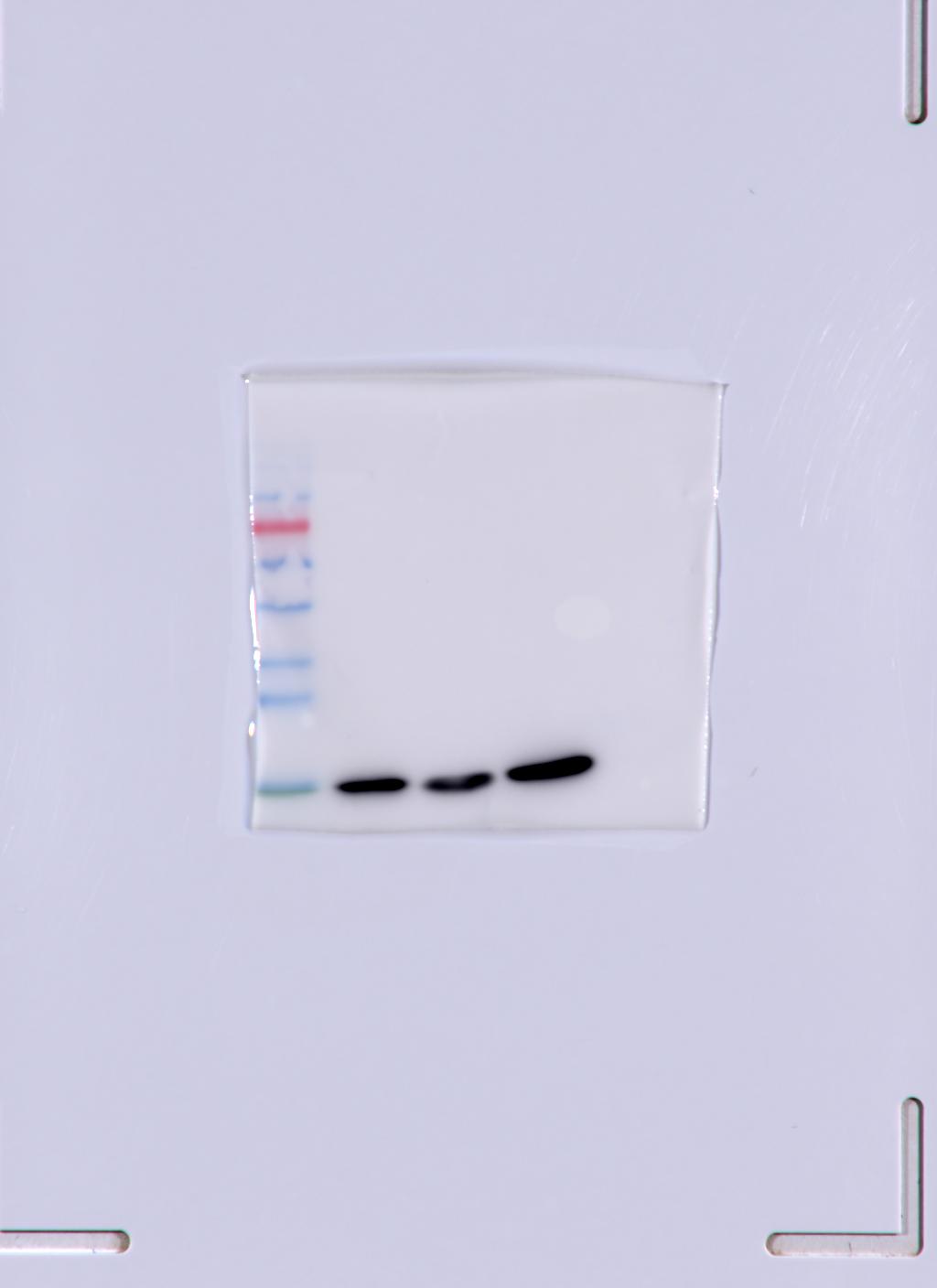


Figure 5 PPARγ Figure 5 C/EBPα


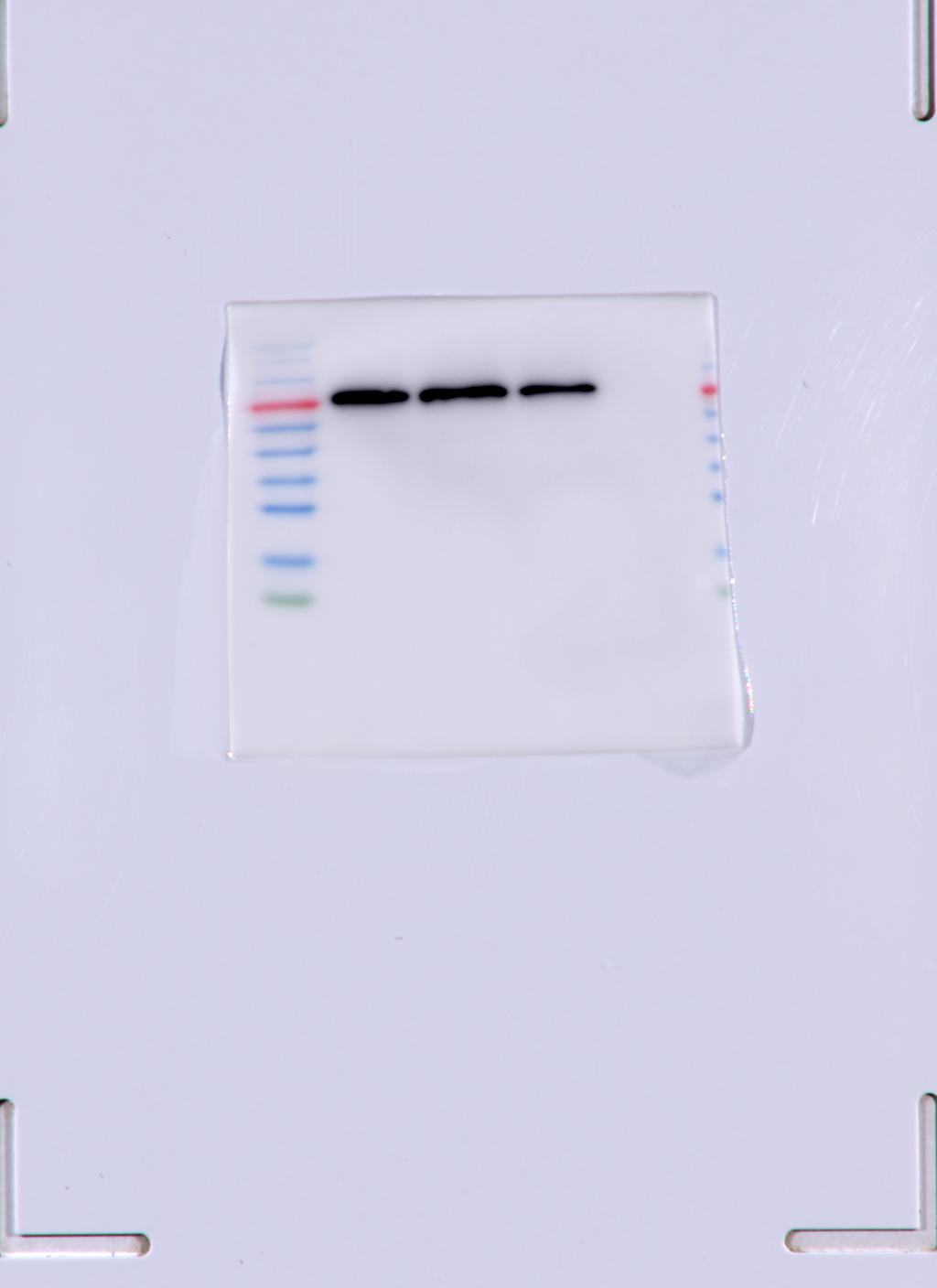

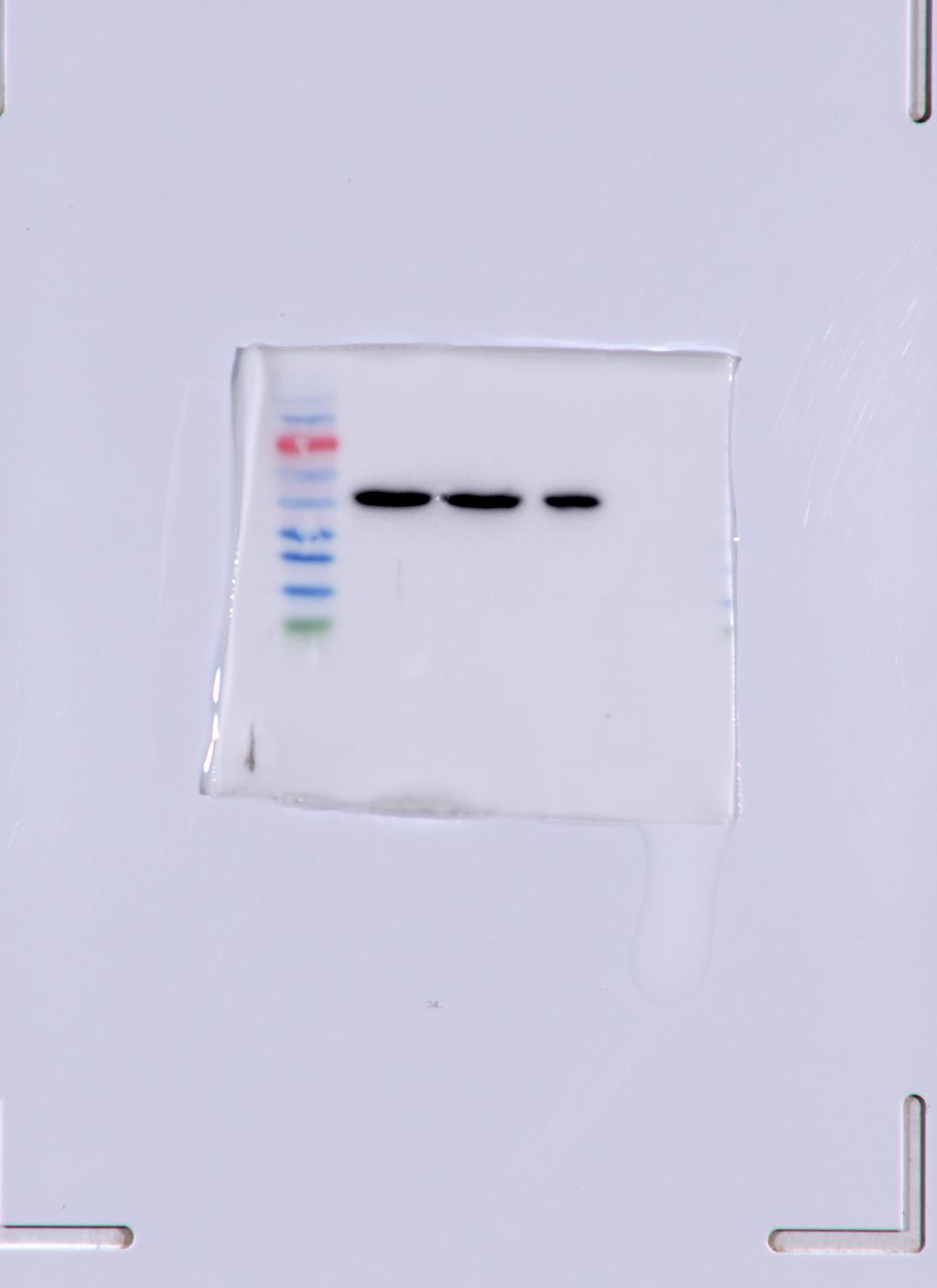


Figure 5 β-action


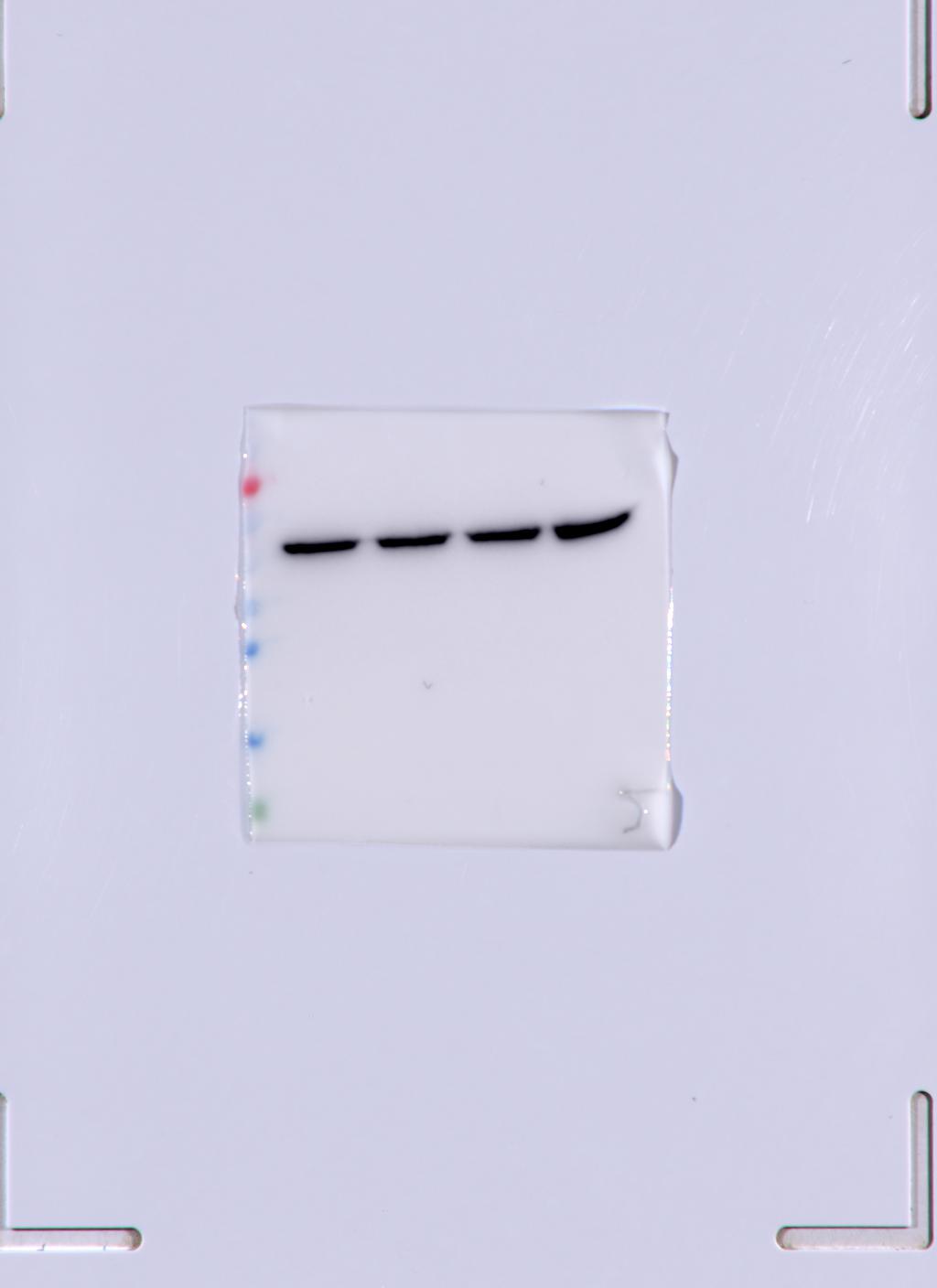

Supplement: Supplementary file 1 — Supplementary Material 1 [file 12891_2024_7481_MOESM1_ESM.docx]
